# Modification Patterns of DNA Methylation-Related lncRNAs Regulating Genomic Instability for Improving the Clinical Outcomes and Tumour Microenvironment Characterisation of Lower-Grade Gliomas

**DOI:** 10.3389/fmolb.2022.844973

**Published:** 2022-03-10

**Authors:** Aierpati Maimaiti, Yirizhati Aili, Mirzat Turhon, Kaheerman Kadeer, Paziliya Aikelamu, Zhitao Wang, Weiwei Niu, Maimaitili Aisha, Maimaitijiang Kasimu, Yongxin Wang, Zengliang Wang

**Affiliations:** ^1^ Department of Neurosurgery, Neurosurgery Centre, The First Affiliated Hospital of Xinjiang Medical University, Urumqi, China; ^2^ Department of Neurointerventional Surgery, Beijing Neurosurgical Institute, Capital Medical University, Beijing, China; ^3^ Department of Neurointerventional Surgery, Beijing Tiantan Hospital, Capital Medical University, Beijing, China

**Keywords:** biomarker, DNA methylation, genomic instability, long non-coding RNA, tumour microenvironment, lower-grade glioma

## Abstract

**Background:** DNA methylation is an important epigenetic modification that affects genomic instability and regulates gene expression. Long non-coding RNAs (lncRNAs) modulate gene expression by interacting with chromosomal modifications or remodelling factors. It is urgently needed to evaluate the effects of DNA methylation-related lncRNAs (DMlncRNAs) on genome instability and further investigate the mechanism of action of DMlncRNAs in mediating the progression of lower-grade gliomas (LGGs) and their impact on the immune microenvironment.

**Methods:** LGG transcriptome data, somatic mutation profiles and clinical features analysed in the present study were obtained from the CGGA, GEO and TCGA databases. Univariate, multivariate Cox and Lasso regression analyses were performed to establish a DMlncRNA signature. The KEGG and GO analyses were performed to screen for pathways and biological functions associated with key genes. The ESTIMATE and CIBERSORT algorithms were used to determine the level of immune cells in LGGs and the immune microenvironment fraction. In addition, DMlncRNAs were assessed using survival analysis, ROC curves, correlation analysis, external validation, independent prognostic analysis, clinical stratification analysis and qRT-PCR.

**Results:** We identified five DMlncRNAs with prognostic value for LGGs and established a prognostic signature using them. The Kaplan–Meier analysis revealed 10-years survival rate of 10.10% [95% confidence interval (CI): 3.27–31.40%] in high-risk patients and 57.28% (95% CI: 43.17–76.00%) in low-risk patients. The hazard ratio (HR) and 95% CI of risk scores were 1.013 and 1.009–1.017 (*p* < 0.001), respectively, based on the univariate Cox regression analysis and 1.009 and 1.004–1.013 (*p* < 0.001), respectively, based on the multivariate Cox regression analysis. Therefore, the five-lncRNAs were identified as independent prognostic markers for patients with LGGs. Furthermore, GO and KEGG analyses revealed that these lncRNAs are involved in the prognosis and tumorigenesis of LGGs by regulating cancer pathways and DNA methylation.

**Conclusion:** The findings of the study provide key information regarding the functions of lncRNAs in DNA methylation and reveal that DNA methylation can regulate tumour progression through modulation of the immune microenvironment and genomic instability. The identified prognostic lncRNAs have high potential for clinical grouping of patients with LGGs to ensure effective treatment and management.

## Introduction

Lower-Grade Gliomas (LGGs) are highly common primary malignancies that affect the central nervous system and are associated with high disability and mortality rates (Louis et al., 2016; Guerreiro Stucklin et al., 2019). The World Health Organization (WHO) classification system of histologically integrated pathological phenotypes and genetic features states that LGGs are classified as grade II and III (Xie et al., 2020). Approximately 70% of patients with LGGs have isocitrate dehydrogenase1 (IDH1) mutations and combined deletion/non-deletion of chromosome 1p19q (Molloy et al., 2020). Patients with combined 1p/19q deletion and IDH mutations have a better prognosis (Park and Turcan, 2019), with a median overall survival (OS) of 8 years. However, patients who do not have 1p/19q deletion (astrocytoma) but have IDH mutations have a median OS of 6.4 years. Patients with LGGs with wildtype IDH have a median OS of 1.7 years, which is similar to the survival time of patients with glioblastoma with wild-type IDH (Pallud et al., 2013; Paľa et al., 2019). Although the prognosis of patients with LGGs is better than that of patients with high-grade gliomas (HGGs), a majority of patients with LGGs have a high risk of advancing to HGGs or recurrence during the development and progression of LGGs, thus leading to higher malignancy and aggressiveness (Weng and Salazar, 2021). Currently available major LGG therapies include chemotherapy, radiotherapy and surgical resection. However, conventional therapies do not significantly improve the prognosis of patients with LGGs. Therefore, it is necessary to identify new biomarkers that can help to improve the early clinical diagnosis of LGG, to assess the potential underlying mechanisms of LGG progression and to develop novel treatment strategies.

Gliomagenesis of LGGs is characterised by significant changes in oncogenes. It may increase the tendency of cells to acquire DNA mutations after dysregulation of mechanisms that maintain genomic integrity, which is known as genomic instability (Kang et al., 2021). Genomic instability is considered a key feature of cancer and is a potential marker for the prognosis of patients with tumours (Malihi et al., 2020). Furthermore, it plays a crucial role in aberrant post-transcriptional and transcriptional modulation, regulation of the expression of miRNA genes and post-transcriptional regulation of lncRNAs (McKay, 2014; Majidinia and Yousefi, 2016). It can be determined by exploring molecular signatures. Several studies have explored genomic-instability signatures in different cancer types. For example, Bao et al. (2021) explored plasma extracellular capsule–miRNA signatures related to genomic instability as a predictive factor for a poor prognosis and increased risk of breast cancer. In addition, Geng et al. (2021) reported that mutation-derived lncRNA signatures related to genomic instability have high prognostic potential in lung adenocarcinoma. In addition to genomic instability caused by DNA mutations, dysregulated epigenetic modifications can significantly affect genomic integrity and alter protein expression levels (Bae et al., 2014; Tong et al., 2020). Histone modifications and DNA methylation are major epigenetic mechanisms that play an essential role in genomic instability (Clark et al., 2021).

DNA methylation promotes heterochromatin formation and gene silencing (Torres-Garcia et al., 2020), whereas histone acetylation relaxes the chromatin structure and hence promotes gene transcription (Ferrari et al., 2020). The functions of histone methylation are more diverse, ranging from activation of transcription (K79, K36 and H3K4) to repression (H4K20, K27 and H3K9) (Hsieh and Fischer, 2005; Dietz et al., 2015). In addition, lysine has three different methylation states (mono-, di- and tri-methylated). To form these methylation states, cells remove [lysine demethylases (KDMs)] and add [lysine methyltransferases (KMTs)] methyl groups in specific lysine residues of histones using corresponding enzymes (Cruz-Tapias et al., 2019). DNA methylation inhibitors and S-adenosylmethionine (SAM) can prevent tumour progression and genomic instability or lead to other changes (Sahin et al., 2010). Furthermore, DNA methyltransferases enhance the resistance of pancreatic cancer (PCa) cells to molecular targeting agents and mediate high methylation of the microRNA 34a promoter (Ma et al., 2020). In addition, the proliferation, invasion and migration of PCa cells are inhibited by MCM3AP-AS1 KD through NPY1R upregulation, which is mediated by DNMT1/DNMT3 (A/B) methylation (Li et al., 2020). It has been reported that patients with glioma exhibiting MGMT promoter methylation have a better survival rate than that of patients without these methylation sites (Pinson et al., 2020; Siller et al., 2021). Moreover, MSS tumours with BRAF mutations usually have high methylation levels, suggesting that the poor survival of patients with colorectal cancer with BRAF mutations is attributed to the relationship between high methylation levels and poor prognosis (Pinson et al., 2020). Hypomethylation and hypermethylation of regulatory regions within genes play a similar role in DNA mutations, thus promoting tumour progression (Jin et al., 2011). Furthermore, epigenetic modification of histones modifies the chromatin structure, leading to rearrangement of chromosomes and, eventually, genetic instability (Giese et al., 2017; Cao et al., 2020a). In conclusion, these epigenetic changes modulate checkpoint regulation and regulate cell cycle progression, which ultimately contribute to tumour progression and genomic instability.

lncRNAs are a group of RNA molecules with a length of more than 200 nucleotides (Zheng et al., 2021). They play a critical role in genomic stability, cell proliferation, cell migration, cell survival and gene regulation (Mao et al., 2021). The key biological roles and associated distribution profiles, which are specific to cells and tissues, imply that lncRNAs are potential biomarkers for cancer diagnosis and prognosis. Furthermore, it has been reported that DNA methyltransferase (DNMT1)-related lncRNAs regulate DNA methylation and gene expression in colorectal cancer (Merry et al., 2015). lncRNAs play a key role in modulating DNA methylation; however, the clinical value of lncRNAs regulating DNA methylation in genomic instability in LGGs has not been comprehensively elucidated. In the present study, the data of 529 patients with LGGs were retrieved from TCGA and analysed to screen for DNA methylation-related lncRNAs through unsupervised clustering of the expression levels of 20 regulators of DNA methylation. The expression levels of lncRNAs regulating DNA methylation in genomic instability were evaluated instead of evaluating DNA methylation because DNA methylation may vary in function depending on the genomic context. The results revealed the prognostic significance of DNA methylation-related lncRNAs in patients LGGs. In addition, we identified five DNA methylation-related lncRNAs at the transcriptional and genomic levels, examined their role in the prognosis of patients and assessed their mechanisms of action in mediating tumour progression in genomic instability, which may provide new insights into their impact on the prognosis of LGGs.

## Materials and Methods

### Collection of LGG Datasets and Preprocessing

LGG transcriptomic data [fragments per kilobase of transcript per million mapped reads (FPKM)], copy number variation (CNV), somatic mutation data and data on phenotypic characteristics were retrieved from The Cancer Genome Atlas (TCGA) database (https://portal.gdc.cancer.gov). To re-annotate lncRNA-associated probes in the gene microarray, we downloaded the appropriate lncRNA genomic sequence information from the GENCODE database (GRCh38.gtf, https://www.gencodegenes.org/human/). Furthermore, the sequence information of the microarray probe was used to match with the sequence of lncRNAs to construct an lncRNA expression profile of the re-annotated microarray. Patients with LGGs with missing OS data or survival time less than 30 days were excluded to minimise statistical bias. In addition, we selected three LGG cohorts [CGGA mRNA-seq-693 (sample size: 332, available: 332 ), CGGA mRNA-seq-325 (sample size: 162, available: 332) and GSE16011 (sample size: 80, available: 80)] for external validation. It was worth noting that CGGA mRNA-seq-693 and CGGA mRNA-seq-325 datasets were created by same organization, and the above data sets have similar clinical information publicly available.

### Selection of DNA Methylation Regulators and lncRNAs Related to Them

We searched the literature related to DNA methylation modifications and selected 20 DNA methylation regulators (Meng et al., 2021) to determine different modification profiles of DNA methylation. These regulators included 14 readers (UHRF1, UHRF2, ZBTB33, MBD4, MBD3, MBD2, MBD1, ZBTB4, ZBTB38, SMUG1, NTHL1, TDG, MECP2 and UNG), 3 erasers (TET3, TET2 and TET1) and 3 writers (DNMT3B, DNMT3A and DNMT1). We screened DNA methylation-related lncRNAs through gene expression correlation analysis and selected 2698 DNA methylation-related lncRNAs. The following parameters were used to screen for DNA methylation-related lncRNAs: |Pearson R| > 0.3 and *p* < 0.001.

### Identification of Genomic Instability of DNA Methylation-Related lncRNAs

To assess the association with genome instability-related lncRNAs, we combined the expression profiles of these lncRNAs and somatic mutation profiles using a bioinformatic model for tumour genomes derived from a mutation hypothesis. The model calculates the cumulative number of somatic mutations in every sample and ranks patients based on the number of somatic mutations from the highest to lowest (Bao et al., 2020). We designated 25% of patients with the lowest number of mutations and 25% of patients with the highest number of mutations as genomic stability-like (GS) and genomic instability (GU) groups, respectively. The “limma” R package was used to determine differences between groups by comparing the mean expression of DNA methylation-related lncRNAs using the Wilcoxon rank-sum test. Differentially expressed lncRNAs were selected (|log_2_ Fc filter| > 0.585 and false discovery rate [FDR]-adjusted *p*-value < 0.05) and denoted as DNA methylation-related lncRNAs (DMlncRNAs). Subsequently, we normalised the expression of all DMlncRNAs using Z-score analysis. In addition, hierarchical clustering analysis was performed using the “limma”, “pheatmap” and “sparcl” R packages and the lncRNAs were grouped into two clusters by calculating the Euclidean distance. The cluster with low mutation levels was denoted as a GS-like cluster, whereas the one with high mutation levels was denoted as a GU-like cluster (*p* < 0.05, Mann–Whitney *U* test). Differential analysis of DMlncRNAs in genomic instability is shown in Supplementary Table S1.

### Identification and Validation of the Risk Score Based on DNA Methylation Regulator-Related lncRNAs in LGGs

The whole TCGA dataset was divided into the validation and training sets in a ratio of 3:7 to verify the findings (“caret” package). A signature of DMlncRNAs was constructed using the training set and validated using the validation and TCGA sets. The baseline characteristics of patients in the three cohorts are presented in Table 1.

**TABLE 1:** Clinicopathological characteristics of the patients with LGG in TCGA cohort.

Covariates	Type	Total (n = 468)	Training set (n = 329)	Testing set (n = 139)	*p* value
Age (%)	<40	215 (45.94%)	151 (45.9%)	64 (46.04%)	1^a^
	≥40	253 (54.06%)	178 (54.1%)	75 (53.96%)	
Gender (%)	Female	212 (45.3%)	152 (46.2%)	60 (43.17%)	0.6163^a^
	Male	256 (54.7%)	177 (53.8%)	79 (56.83%)	
Tumor Grade (%)	G2	226 (48.29%)	156 (47.42%)	70 (50.36%)	0.6512^a^
	G3	241 (51.5%)	172 (52.28%)	69 (49.64%)	
	Unknown	1 (0.21%)	1 (0.3%)	0 (0%)	
New tumor event after initial treatment (%)	Yes	128 (27.35%)	92 (27.96%)	36 (25.9%)	0.9050^a^
	No	256 (54.7%)	181 (55.02%)	75 (53.96%)	
	Unknown	84 (17.95%)	56 (17.02%)	28 (20.14%)	
Radiation therapy (%)	Yes	267 (57.05%)	182 (55.32%)	85 (61.15%)	0.2074^a^
	No	153 (32.69%)	114 (34.65%)	39 (28.06%)	
	Unknown	48 (10.26%)	33 (10.03%)	15 (10.79%)	
Diagnoses Type (%)	Astrocytoma,anaplastic	120 (25.64%)	82 (24.92%)	38 (27.34%)	0.9112^b^
	Astrocytoma,NOS	56 (11.97%)	40 (12.16%)	16 (11.51%)	
	Mixedglioma	124 (26.5%)	88 (26.75%)	36 (25.9%)	
	Oligodendroglioma,anaplastic	70 (14.96%)	52 (15.81%)	18 (12.95%)	
	Oligodendroglioma,NOS	98 (20.94%)	67 (20.36%)	31 (22.3%)	
Sample type (%)	Primary Tumor	450 (96.15%)	315 (95.74%)	135 (97.12%)	0.6562^a^
	Recurrent Tumor	18 (3.85%)	14 (4.26%)	4 (2.88%)	
Chr 19/20 co-gain (%)	Gain chr 19/20	11 (2.35%)	8 (2.43%)	3 (2.16%)	1^a^
	No chr 19/20 gain	454 (97.01%)	318 (96.66%)	136 (97.84%)	
	Unknown	3 (0.64%)	3 (0.91%)	0 (0%)	
Chr 7 gain/Chr 10 loss (%)	Gain chr 7 and loss chr 10	52 (11.11%)	35 (10.64%)	17 (12.23%)	0.7586^a^
	No combined CNA	413 (88.25%)	291 (88.45%)	122 (87.77%)	
	Unknown	3 (0.64%)	3 (0.91%)	0 (0%)	
IDH1 R132 status (%)	Mutation	361 (77.14%)	250 (75.99%)	111 (79.86%)	0.4295^a^
	Wild	107 (22.86%)	79 (24.01%)	28 (20.14%)	
IDH2 R172 status (%)	Mutation	18 (3.85%)	15 (4.56%)	3 (2.16%)	0.3315^a^
	Wild	450 (96.15%)	314 (95.44%)	136 (97.84%)	
PTEN status (%)	Mutation	28 (5.98%)	19 (5.78%)	9 (6.47%)	0.9375^a^
	Wild	440 (94.02%)	310 (94.22%)	130 (93.53%)	
EGFR status (%)	Mutation	29 (6.2%)	21 (6.38%)	8 (5.76%)	0.9621^a^
	Wild	439 (93.8%)	308 (93.62%)	131 (94.24%)	
ATRX status (%)	Mutant	173 (36.97%)	120 (36.47%)	53 (38.13%)	0.8148^a^
	WT	295 (63.03%)	209 (63.53%)	86 (61.87%)	
TP53 status (%)	Mutation	217 (46.37%)	146 (44.38%)	71 (51.08%)	0.2198^a^
	Wild	251 (53.63%)	183 (55.62%)	68 (48.92%)	

a Chi square test.

b Wilcoxon rank sum test.

The relationship between the OS of patients and expression levels of DMlncRNAs were examined through univariate Cox proportional risk regression analysis using LGG survival data retrieved from TCGA (*p* < 0.05). The “glmnet” R package was used for Lasso Cox regression analysis (with 1,000 iterations) to identify DMlncRNAs associated with the OS of patients with LGGs. Finally, the risk coefficients of prognostic DMlncRNAs were determined *via* multivariate Cox proportional risk regression analysis. A prediction model (DMlncRNA risk score) was constructed based on the expression levels of these prognostic DMlncRNAs and the coefficients evaluated *via* multivariate regression analysis, using the following formula:

$$\text{DMlncRNA Riskscore}=\sum_{\text{i}=1}^{\text{n}}{\text{coefDMlncRNA}}_{i}\times {\text{ExprDMlncRNA}}_{i}$$

In this formula, coef represents the coefficient in multivariate Cox regression analysis, ExprDMlncRNAi represents the expression level of lncRNAs and coefDMlncRNAi represents the coefficients of lncRNAs associated with survival. Patients with LGGs were divided into the low- and high-risk groups according to the median DMlncRNA risk score as the cut-off value. Survival curves were generated for both groups using the Kaplan–Meier method, and logarithmic tests were performed using the ‘survminer’ and ‘survival’ R packages. A *p*-value < 0.05 was considered statistically significant. In addition, time-dependent receiver operating characteristic (ROC) curves were generated using the ‘survivalROC’ R package to examine the prognostic significance of the risk score. The validation and TCGA cohorts were used to validate the DMlncRNA risk signature.

### Evaluation of the Independent Prognostic Value of the Risk Score Based on DNA Methylation Regulator-Related lncRNAs

Clinical data from the training, test and TCGA cohorts and risk scores were used for univariate and multivariate Cox regression analyses to assess the clinical value of the risk score as an independent prognostic marker. The area under the ROC curve (AUC) was determined to evaluate the accuracy of the prognostic signature using the “pROC” R package.

### Clinical Stratification Analysis and Analysis of the Prognostic Value of the DMlncRNA Risk Score

Univariate and multivariate Cox regression analyses were performed for each variable in the training, test and TCGA cohorts using the “survivor” R package to determine whether the DMlncRNA risk score was an independent prognostic marker for other important clinicopathological characteristics. A *p*-value < 0.05 was considered statistically significant. The reliability of the DMlncRNA risk score in predicting prognosis was determined through clinical stratification analysis. Patients in the TCGA cohort were assigned to subclasses based on the following clinical characteristics: tumour stage (stage II and III), sex (female and male), history of radiation therapy, tumour type (primary and recurrent) and age (≥40 and <40 years). Patients in each clinical subgroup were divided into the low- and high-risk groups based on the median risk score. Survival differences between the high- and low-risk groups in the subgroups were compared using the log-rank test and Kaplan–Meier analysis.

### Establishment and Verification of a Prognostic Nomogram for LGGs

A nomogram was constructed based on the clinical characteristics of DMlncRNAs and the risk score to improve the prognostic value. The “rms” R package was used to generate column line plots for independent prognostic factors and relevant clinical parameters as variables in the training and validation cohorts. Points for each variable were indicated using a horizontal line according to the different variable characteristics. The total number of points for every patient was determined by calculating the sum of points for each value, and the values were normalised to a range of 0–100. The 1-, 3- and 5-years OS of patients with LGGs were calculated by placing them between each prognostic axis and the total score axis. The ‘survcomp’ and ‘rms’ R packages were used to generate calibration plots based on the concordance index (C-index) and its 95% confidence interval (CI). Clinical decision curve analysis (DCA) was conducted using the ‘rmda’ and ‘devtools’ R packages to verify the performance of the column line plots in the validation and training cohorts.

### Validation of the Risk Score Based on DNA Methylation Regulator-Related lncRNAs Using an External Cohort

The GSE16011 dataset retrieved from the Gene Expression Omnibus (GEO) database and the mRNAseq-325 and mRNAseq-693 datasets retrieved from the China Glioma Genome Atlas (CGGA) database were used to validate the DMlncRNA signature. The same formula (DMlncRNA risk score) was used to calculate the risk score, and a boxplot was generated to compare gene expression levels based on the following characteristics: chromosome 1p/19q combined deletion status, chemotherapy status, IDH1 mutation status, tumour type (primary and recurrent), tumour grade and age.

### Evaluation of the Tumour Microenvironment and Immune Cell Infiltration in LGGs

Immune, stromal and ESTIMATE scores were evaluated using the ESTIMATE algorithm, and the relationship between the tumour microenvironment and risk scores was examined. The correlation between the OS of patients with LGGs and algorithm scores were evaluated using Kaplan–Meier analysis. The CIBERSORT algorithm was used to determine the proportion of 22 immune cells using the gene expression data of LGG patients to further evaluate differences in immune cell infiltration between the high- and low-risk groups. Only data with a CIBERSORT *p* value <0.05 was filtered and reserved for the following analysis. The output was directly integrated to generate an entire matrix of immune cell fractions. The levels of immune cells with significantly different proportions in the high- and low-groups were determined using the Wilcoxon rank-sum test. The correlation between the OS of patients with LGGs and levels of the 22 infiltrating immune cells was determined through Kaplan–Meier analysis. In addition, the correlation between immune cells and risk scores was evaluated through Pearson correlation analysis.

### KEGG Pathway Enrichment Analysis and Gene Oncology Annotation

The “clusterProfiler” R package was used to perform KEGG and GO pathway enrichment analyses. Statistical significance was indicated by *p*-value < 0.05.

### RNA Extraction and qRT-PCR

From June 2020 to June 2021, 16 LGG and adjacent normal brain samples were obtained from eight patients who had undergone surgical dissection and pathological confirmation at the First Affiliated Hospital of Xinjiang Medical University. The present study was approved by the Medical Research Ethics Committee of the First Affiliated Hospital of Xinjiang Medical University. An RNA reagent (Wuhan Servicebio Technology Co., LTD, Wuhan, China) was used to extract total RNA. NanoDrop 2000 (Thermo Fisher Scientific, Waltham, MA, United States) was used to determine RNA quantity. mRNA levels were quantified using a two-step reaction process, namely, reverse transcription (RT) polymerase chain reaction (PCR). Servicebio RT First Strand cDNA Synthesis Kit (Wuhan Servicebio Technology Co., LTD, Wuhan, China) was used to synthesise cDNA from RNA. The expression levels of GAPDH, colorectal neoplasia differentially expressed (CRNDE), CYTOR, MPPED2-AS1 and SNHG18 were evaluated through qRT-PCR using the SYBR Green qPCR Master Mix (High ROX) (Servicebio, Wuhan, China). The expression levels were evaluated relative to the GAPDH expression level. The following PCR primer sequences were obtained from Servicebio (Wuhan): GAPDH-F: 5′-GGA​AGC​TTG​TCA​TCA​ATG​GAA​ATC-3′, GAPDH-R: 5′-TGA​TGA​CCC​TTT​TGG​CTC​CC-3′; CRNDE-F: 5′-GGA​AAA​ATC​AAA​GTG​CTC​GAG​TG-3′, CRNDE-R: 5′-ACT​GGC​AAT​CAA​ATA​CAG​CTT​AAC​C-3′; CYTOR-F: 5′-AAA​ATC​ACG​ACT​CAG​CCC​CC-3′, CYTOR-R: 5′-AAT​GGG​AAA​CCG​ACC​AGA​CC-3′; SNHG18-F: 5-GGA​GCC​ACC​CAG​AAA​CTT​AGA​CA-3′, SNHG18-R: 5-CCC​TGG​TGG​ACT​TGA​GTG​GAA-3′ and MPPED2-AS1-F: 5′-TAG​AAA​CAC​CCC​TTC​GGA​AAC​AC-3′, MPPED2-AS1-R: 5′-CCT​TTG​GTG​ACC​TTA​TCT​AGT​TAC​TGA -3′. The expression levels of CRNDE, CYTOR, MPPED2-AS1 and SNHG18 were determined using the 2^(−ΔΔCT)^ method. The amplification reaction included the following steps: pre-denaturation at 95°C for 10 min, followed by denaturation at 95°C for 40 cycles of 15 s and extension at 60°C for 30 s. Fluorescence signals were recorded from 65 to 95°C at an interval of 0.3°C.

### Evaluation of Potential Candidate Drugs

Connectivity Map (cMap), a gene expression profiling database, was used to screen for potential drug compounds against LGGs. We uploaded differentially expressed DMlncRNAs to the database to identify potential connections and bioactive compounds. The linkage score was set between -1 and 1 to assess the closeness of the active agent associated with the query feature. A positive score implied that the drug promoted the expression of high-risk lncRNAs, whereas a negative score implied that the drug inhibited the expression of high-risk lncRNAs. A threshold of *p*-value < 0.05 was set to indicate significance.

### Statistical Analysis

R (version 4.0.3) and Perl tools were used to conduct all statistical analyses. Continuous data were analysed using the Wilcoxon test, and categorical variables were analysed using the Fisher’s exact or chi-square test. Survival differences were estimated using the KM and log-rank tests. In addition, differential expression analyses were performed for LGG (N = 518) and matched normal samples obtained from TCGA and samples obtained from Genotype-Tissue Expression (GTEx) (N = 207) through Gene Expression Profile Interaction Analysis (GEPIA) (http://gepia.cancer-pku.cn/). A *p*-value < 0.05 indicated statistical significance.

## Results

### DNA Methylation Regulator-Related lncRNAs Were Identified in Patients With LGGs

**FIGURE 1:**
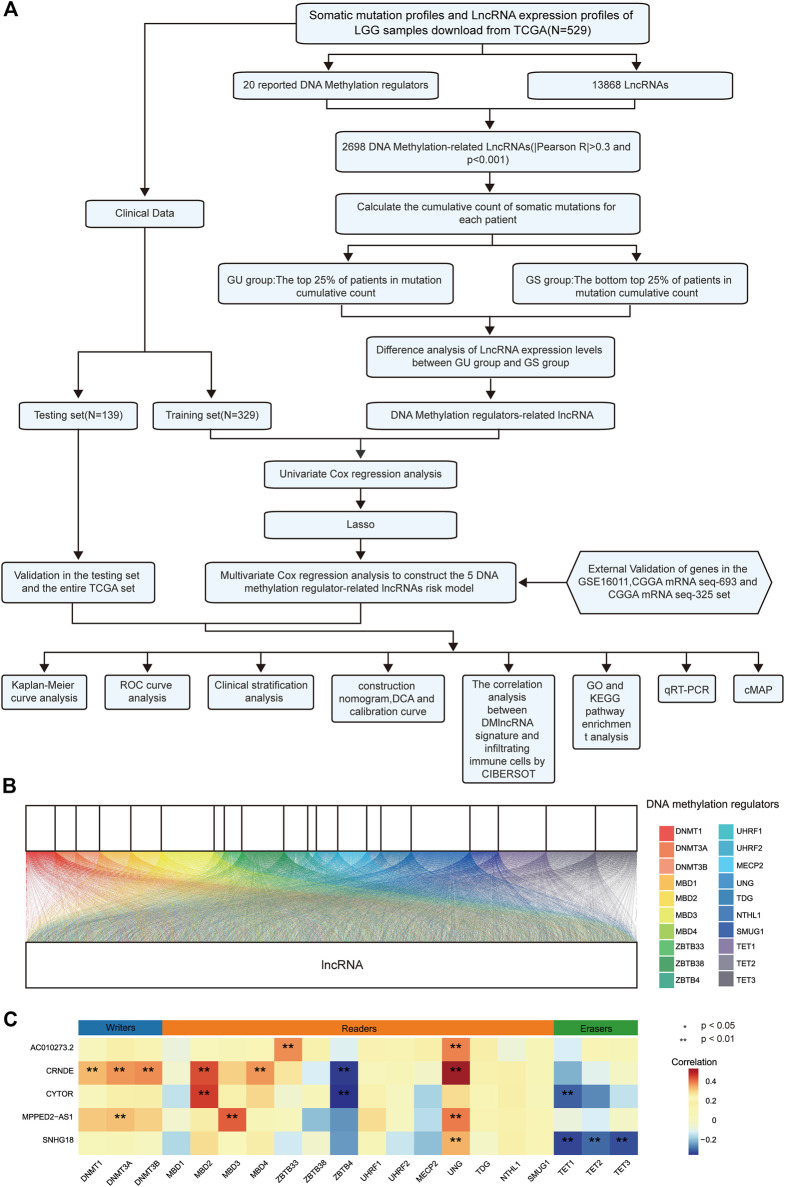
DNA methylation-related lncRNAs (DMlncRNAs) in patients with LGGs. **(A)** Flow chart of the study design. **(B)** Sankey relational diagram for 20 regulatory genes of DNA methylation and DMlncRNAs. **(C)** Heat map representing the correlation among the 20 DNA methylation-related genes and the 5 prognostic DMlncRNAs.

A schematic illustration of the construction of the DMlncRNA prognostic signature and subsequent analyses is presented in Figure 1A. We extracted the TCGA cohort matrix comprising 13,868 lncRNAs and evaluated the expression levels of 20 DNA methylation-related genes. The lncRNAs highly associated with any of the 20 DNA methylation-associated genes were identified as DMlncRNAs (|Pearson R| > 0.3 and *p* < 0.001). A Sankey plot was generated to visualise the DMlncRNA co-expression network (Figure 1B), and a total of 2,698 DMlncRNAs were identified. Figure 1C shows the correlation between DNA methylation-associated genes and DMlncRNAs in the TCGA set.

### Regulation of Genomic Instability *via* DNA Methylation Regulator-Related lncRNAs in LGGs

The total number of somatic mutations was calculated for all patients, and the patients were ranked in descending order to determine the genomic instability of DMlncRNAs. The bottom 25% (*n* = 137) and top 25% (*n* = 133) patients were divided into the GU- and GS-like groups, respectively. A total of 165 differentially expressed lncRNAs were identified as novel DMlncRNAs by comparing the significantly different lncRNA expression profiles of patients in the GS- and GU-like groups. Of the 165 lncRNAs, 85 were downregulated and 80 were upregulated (log FC filter| > 0.585, FDR-adjusted *p*-value < 0.05; Wilcoxon test) (Supplementary Table S1). A heat map was generated based on the top 20 downregulated and upregulated lncRNAs (Supplementary Figure S1A). A total of 529 samples in the TCGA set were used for unsupervised hierarchical cluster analysis based on the expression levels of the 165 differentially expressed DMlncRNAs, and patients were divided into the GU- and GS-like groups (Supplementary Figure S1B). The findings showed that some lncRNAs expression were significantly different between the two groups.

### Establishment of a Prognostic DNA Methylation Regulator-Related lncRNA Signature Using the Training Cohort

**FIGURE 2:**
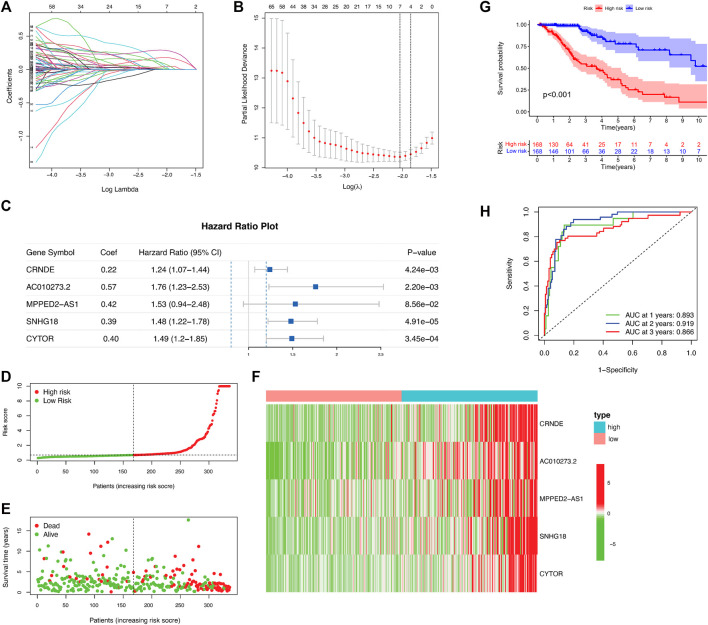
DNA methylation-related lncRNA (DMlncRNA) signature for predicting outcomes in the training set. **(A)** Lasso–Cox analysis suggests a significant correlation between the five DMlncRNAs and patient survival. **(B)** Optimal values of penalty parameters determined *via* cross-validation with 1,000 replicates. **(C)** Multivariate Cox regression analysis suggests a high correlation between the selected DMlncRNAs and clinical prognosis. **(D)** Distribution of the DMlncRNA model-based risk signature. **(E)** Various profiles of survival time and survival status in the low- and high-risk groups. **(F)** Heat map showing the expression levels of the five prognostic lncRNAs in all patients. **(G)** The overall survival of high- and low-risk patients in the training cohort determined based on DNA methylation-related lncRNAs *via* Kaplan–Meier analysis. **(H)** 1-, 2- and 3-years ROC curves of patients.

A total of 468 patients with LGGs in TCGA were divided into the test set (*n* = 139) and training (*n* = 329) sets to assess the predictive role of the selected DMlncRNAs in prognosis. Univariate Cox analysis was conducted to examine the association between the expression of 165 DMlncRNAs and the OS of patients in the training set to screen for prognosis-related DMlncRNAs. The results revealed that 132 DMlncRNAs were highly correlated with the prognosis of patients with LGGs (*p* < 0.001). Lasso regression analysis was conducted on these lncRNAs to minimise overfitting. Lasso regression is a commonly used multiple regression analysis used to fit generalised linear models while performing variable screening and complexity adjustment and enables simultaneous variable selection and regularisation. It is widely used to optimise feature selection with low correlation and prominent predictive values in high-dimensional data. Therefore, it can accurately discriminate among most predictive markers and help to identify prognostic indicators for the effective prediction of clinical outcomes. The first rank value of log λ with a minimum segment likelihood deviation was represented as a dashed vertical line. Subsequently, we identified seven lncRNAs involved in DNA methylation in LGGs (Figure 2A) and determined the optimum value of the threshold *via* 1,000 rounds of iterations (Figure 2B). Furthermore, stepwise multivariate Cox regression analysis of these seven candidate lncRNAs identified five DMlncRNAs as prognostic risk factors (Figure 2C and Table 2), including CRNDE, AC010273.2, MPPED2-AS1, SNHG18 and CYTOR. We constructed a risk score based on these DMLncRNAs (DMlncRNA risk score) to assess the risk of prognosis of patients with LGGs based on the expression levels of these five independent prognosis-related DMlncRNAs and multifactorial Cox coefficients. The formula for calculating the risk score is as follows: DMlncRNA Riskscore = [CRNDE ∗ 0.2160] + [AC010273.2 ∗ −0.5662] + [MPPED2-AS1 ∗ 0.4249] + [SNHG18 ∗ 0.3898] + [CYTOR ∗ 0.3979].

**TABLE 2:** Multivariate Cox regression analysis of the five lncRNAs in DMlncRNA model.

LncRNA	Coefficient	HR	HR.95%Low	HR.95%High	*p* value
CRNDE	0.216,023	1.241,131	1.070351	1.439,161	0.004235
AC010273.2	0.566,201	1.761,563	1.225,988	2.531,104	0.002201
MPPED2-AS1	0.424,854	1.529,368	0.942,137	2.482,617	0.085646
SNHG18	0.389,813	1.476,704	1.223,391	1.782,468	4.91E-05
CYTOR	0.397,938	1.488,751	1.197,235	1.851,250	0.000345

We first calculated the risk score of all patients in the training cohort, and patients were divided into the low- and high-risk groups using the median risk score as the threshold. Furthermore, we examined the relationship between the expression of the five DMlncRNAs and OS status and risk score in the training and test cohorts (Figures 2D–F), and the heat map indicated that the five DMlncRNAs were significantly upregulated in the high-risk group. Kaplan–Meier analysis revealed significantly better survival outcomes (OS) for low-risk patients than for high-risk patients, indicating that the prognostic risk score was reliable (*p* < 0.001) (Figure 2G). The survival analysis curves revealed that the 3-, 5- and 10-years survival rates of high-risk patients were approximately 53.6% (95% CI: 44.57–64.5%), 34.62% (95% CI: 24.76–48.4%) and 5.55% (95% CI: 0.98–31.4%), respectively, whereas those of low-risk patients were approximately 91.0% (95% CI: 85.0–97.4%), 77.9% (95% CI: 67.5–90.0%) and 52.3% (95% CI: 35.1–78.0%), respectively. The AUC values of the ROC curves of the DMlncRNA signature for predicting 1-, 2- and 3-years OS were 0.893, 0.919 and 0.866, respectively, (Figure 2H).

### Validation of the Prognostic DNA Methylation Regulator-Related lncRNA Signature in the TCGA and Test Cohorts

**FIGURE 3:**
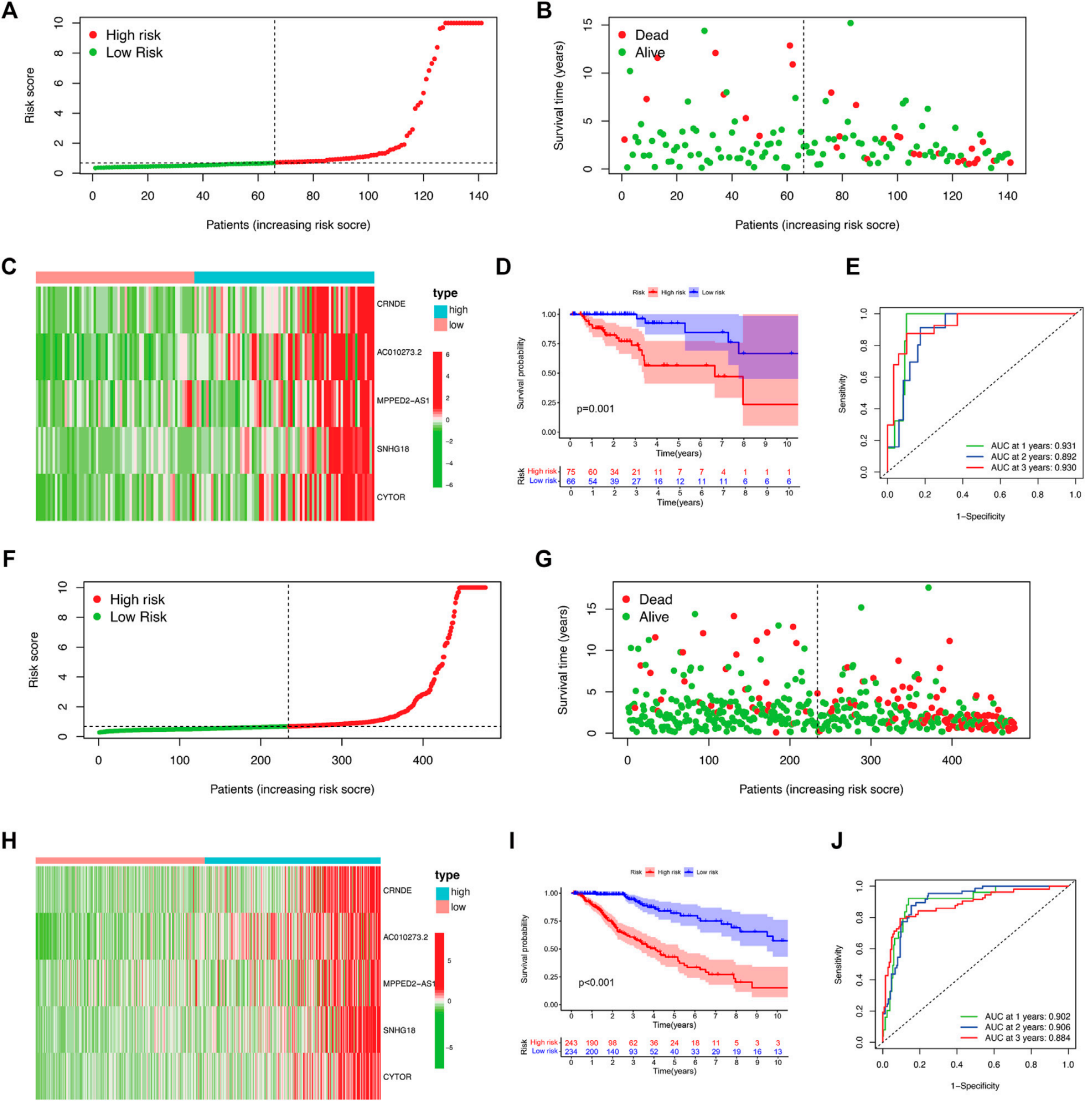
Validation of the 5-DNA-methylation-related-lncRNA (DMlncRNA) risk model in the test and TCGA cohorts. **(A)** DMlncRNA model-based risk signature profile in the test cohort. **(B)** Survival status and survival time profiles in the low- and high-risk groups in the test cohort. **(C)** Heat map showing the expression profiles of the five prognostic DMlncRNAs for each patient in the test cohort. **(D)** Overall survival of patients in the low- and high-risk groups based on Kaplan–Meier analysis in the test cohort. **(E)** ROC curves for predicting 1-, 2- and 3-years survival rates based on DMlncRNAs in the test cohort. **(F–J)** Validation of these findings in TCGA cohort.

A test set including 139 patients was used to validate the prognostic value of the DMlncRNA signature. Using the threshold risk score of the training cohort, the 139 patients in the test set were divided into the high- and low-risk groups. We generated risk curves, scatter plots and heat maps to demonstrate the relationship between risk scores and the OS of patients with LGGs in the test cohort (Figures 3A–C). Similar to the OS of patients in the training set, the OS of high-risk patients in the test set was significantly poorer than that of low-risk patients as demonstrated by Kaplan–Meier curves (*p* < 0.001) (Figure 3D). Furthermore, the 3-, 5- and 10-years survival rates of high-risk patients were approximately 69.6% (95% CI: 56.66–85.5%), 47.1% (95% CI: 29.31–75.8%), and 23.6% (95% CI: 5.45–98.6%), respectively, whereas the 3-, 5- and 10-years survival rates of low-risk patients were approximately 96.3% (95% CI: 69.21–100%), 84.7% (95% CI: 89.43–100%) and 53.4% (95% CI: 29.69–96.0%), respectively. The 1-, 2- and 3-years ROC curves for the DMlncRNA model showed AUC values of 0.931, 0.892 and 0.930 (Figure 3E). The prognostic performance of DMlncRNAs in TCGA cohort was consistent with that in the test cohort. Patients in TCGA cohort were divided into the low- and high-risk groups, and the results of risk curves, scatter plots and heat maps for the association between risk scores and the survival status of patients with LGGs in TCGA cohort were consistent with those in the training and validation sets (Figures 3F–H). Similarly, the survival curves revealed that the OS of the low-risk group was significantly higher than that of the high-risk group (*p* < 0.001) (Figure 3I). The 3-, 5- and 10-years survival rates of high-risk patients were approximately 59.9% (95% CI: 52.39–68.4%), 41.0% (95% CI: 32.02–52.4%) and 10.1% (3.27–31.4%), respectively, whereas those of low-risk patients were approximately 93.51% (95% CI: 89.12–98.1%), 79.86% (95% CI: 70.98–89.9%) and 57.28% (95% CI: 43.17–76.0%), respectively. In addition, the AUC values for predicting 1-, 2- and 3-years OS were 0.902, 0.906 and 0.884, respectively, (Figure 3J).

### Verification and Identification of the Clinical Features of the DNA Methylation Regulator-Related lncRNAs Signature in LGGs

**FIGURE 4:**
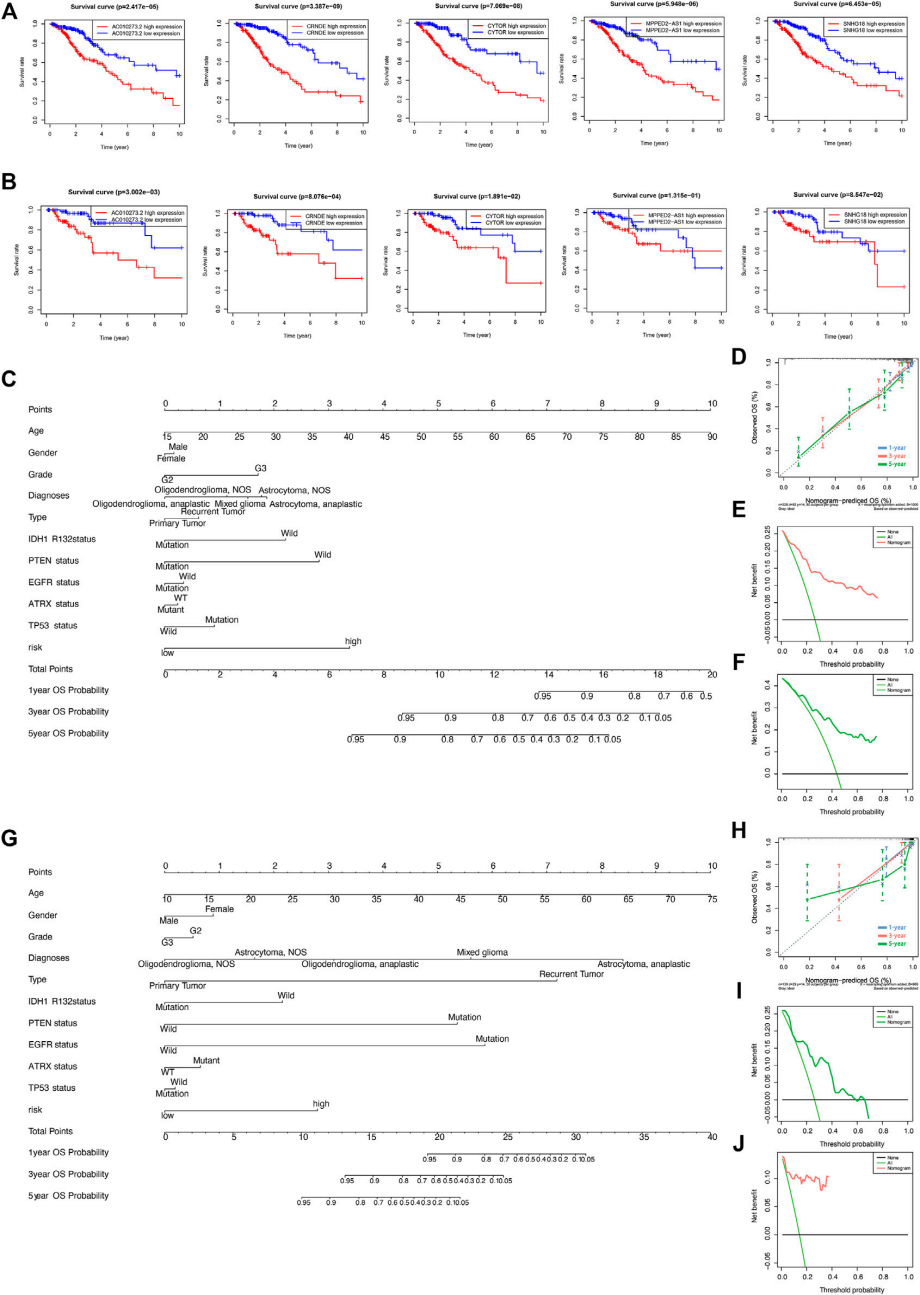
**(A,B)** Prognostic value of the five DNA methylation-related lncRNAs (DMlncRNAs) in the training **(A)** and test **(B)** cohorts. **(C–F)** Nomogram based on the five DMlncRNAs for every feature in the training cohort to predict 1-, 3- and 5-years survival. Nomograms were verified *via* DCA and calibration curves. **(G–J)** Nomogram based on the five DMlncRNAs for every feature in the test cohort to predict 1-, 3- and 5-years survival and verification of nomogram *via* DCA and calibration curves.

Kaplan–Meier curves were generated to predict the OS of LGGs based on the five DMlncRNAs in the training cohort to evaluate their prognostic value (Figure 4A). The five lncRNAs, namely, AC010273.2, CRNDE, CYTOR, MPPED2-AS1 and SNHG18, were associated with OS (*p* < 0.001) and identified as risk factors because their upregulation was correlated with a poor prognosis. Similar results were obtained in the validation set (Figure 4B). Subsequently, we constructed a nomogram based on these DMlncRNAs in the training set and included the following factors: age, ATRX mutation status, sex, PTEN mutation status, tumour grade, diagnosis type, tumour staging, IDH1 R132 mutation status, EGFR mutation status, TP53 mutation status and the risk scores of the five DMlncRNAs (Figure 4C). The nomogram was used to predict the 1-, 3- and 5-years survival rates of patients. A C-index of 0.8439 (95% CI: 0.8055–0.8823) was obtained, which verified the predictive value of the nomogram. In addition, a calibration plot (Figure 4D) and clinical decision curve (Figure 4E) demonstrated that the column line plot had an excellent prediction value. The prediction value of the column line plot was higher than that of the risk score. The findings of the validation cohort were consistent with those of the training cohort (Figures 4F–H).

### Assessment of the Independence of the DNA Methylation Regulator-Related lncRNA Signature in Prognostic Prediction

**FIGURE 5:**
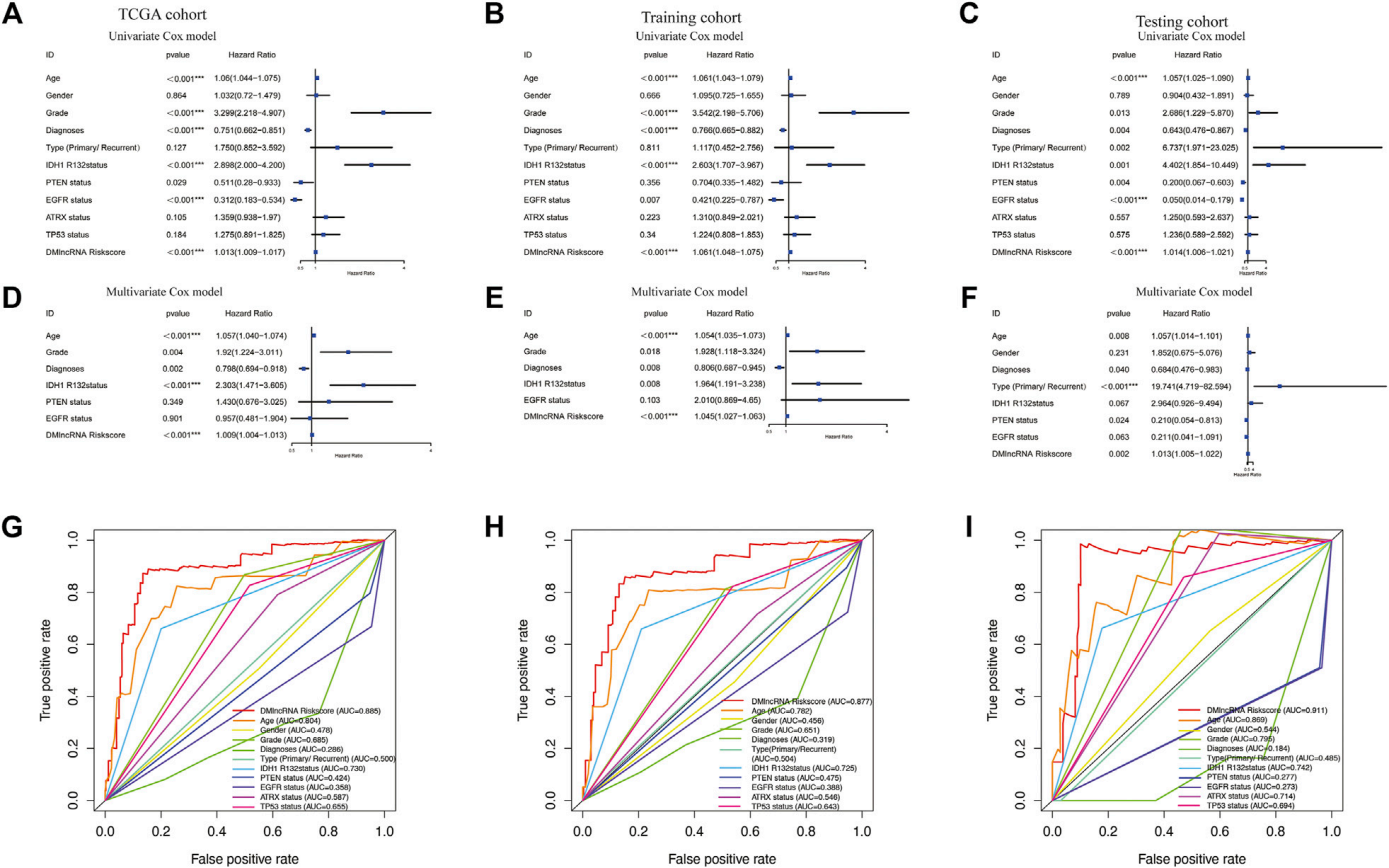
Validation of DNA methylation-related lncRNAs (DMlncRNAs) as an independent prognostic biomarker. **(A–C)** Sex, grade, age, diagnoses, tumour type (primary/recurrent), IDH1 R132 mutation status, PTEN status, EGFR status, ATRX status, TP53 status and DMlncRNAs are accurate markers for predicting the prognosis of patients with LGGs. **(D–F)** Multivariate Cox regression analysis suggests that DMlncRNAs and age are correlated with overall survival, thus indicating that DMlncRNAs are independent predictors of survival in patients with LGGs. **(G–I)** ROC curve analysis suggests high prognostic accuracy of clinicopathological parameters, such as age, sex, tumour grade, diagnoses, tumour type (primary/recurrent), IDH1 R132 mutation status, PTEN status, EGFR status, ATRX status, TP53 status and DMlncRNA-based prognostic risk score, in predicting 1-, 3- and 5-years survival.

Tumour type (primary and recurrent), tumour grade, sex, age, LGG diagnosis type, TP53 mutation status, IDH1 R132 mutation status, EGFR mutation status, ATRX mutation status and PTEN mutation status were used to evaluate the independence of the DMlncRNA signature in predicting prognosis. In addition, multivariate and univariate Cox regression analyses were conducted. The findings of multivariate analysis indicated that the DMlncRNA risk score was highly correlated with the OS of patients in the training, test and TCGA cohorts (*p* < 0.05), and LGG diagnosis type and age were significantly correlated with OS (*p* < 0.05) as well as the DMlncRNA risk score (Table 3 and Figures 5A–F). Furthermore, ROC curves were generated to compare the DMlncRNA risk score with the clinical characteristics of patients. The findings revealed that the risk score was better than tumour type (primary and recurrent), tumour grade, sex, age, LGG diagnosis type, PTEN mutation status, IDH1 R132 mutation status, EGFR mutation status, ATRX mutation status and TP53 mutation status (Figures 5G–I). These findings suggested that the performance of the DMlncRNA risk score in predicting the survival of patients with LGGs was significantly higher.

**TABLE 3:** Univariate and Multivariate Cox Proportional-Hazards analysis for the DMlncRNA Riskscore and overall survival in different LGG patient cohorts.

Variables		Univariable analysis			Multivariable analysis		
		HR	95%CI	*p*-value	HR	95%CI	*p*-value
Training cohort							
DMlncRNA Riskscore	High/Low	1.061	1.048–1.075	<0.001	1.045	1.027–1.063	<0.001
Age		1.061	1.043–1.079	<0.001	1.054	1.035–1.073	<0.001
Gender	Female/Male	1.095	0.725–1.655	0.666	—	—	—
Grade	G2/G3	3.542	2.198–5.706	<0.001	1.928	1.118–3.324	0.018
Diagnoses	Astrocytoma, anaplastic/Astrocytoma, NOS/Mixed glioma/Oligodendroglioma, anaplastic/Oligodendroglioma, NOS	0.766	0.665–0.882	<0.001	0.806	0.687–0.945	0.008
Type	Primary/Recurrent	1.117	0.452–2.756	0.811	—	—	—
IDH1 R132status	Mutation/Wild	2.603	1.707–3.967	<0.001	1.964	1.191–3.238	0.008
EGFR status	Mutation/Wild	0.421	0.225–0.787	0.007	2.010	0.869–4.650	0.103
ATRX status	Mutation/Wild	1.310	0.849–2.021	0.223	—	—	—
TP53 status	Mutation/Wild	1.224	0.808–1.853	0.340	—	—	—
PTEN status	Mutation/Wild	0.704	0.335–1.482	0.356	—	—	—
Testing cohort							
DMlncRNA Riskscore	High/Low	1.014	1.006–1.021	<0.001	1.013	1.005–1.022	0.002
Age		1.057	1.025–1.090	<0.001	1.057	1.014–1.101	0.008
Gender	Female/Male	0.904	0.432–1.891	0.789	—	—	—
Grade	G2/G3	2.686	1.229–5.870	0.013	1.852	0.675–5.076	0.231
Diagnoses	Astrocytoma, anaplastic/Astrocytoma, NOS/Mixed glioma/Oligodendroglioma, anaplastic/Oligodendroglioma, NOS	0.643	0.476–0.867	0.004	0.684	0.476–0.983	0.040
Type	Primary/Recurrent	6.737	1.971–23.025	0.002	19.741	4.719–82.594	<0.001
IDH1 R132status	Mutation/Wild	4.402	1.854–10.449	0.001	2.964	0.926–9.494	0.067
EGFR status	Mutation/Wild	0.050	0.014–0.179	<0.001	0.211	0.041–1.091	0.063
ATRX status	Mutation/Wild	1.250	0.593–2.637	0.557	—	—	—
TP53 status	Mutation/Wild	1.236	0.589–2.592	0.575	—	—	—
PTEN status	Mutation/Wild	0.200	0.067–0.603	0.004	0.210	0.054–0.813	0.024
TCGA cohort							
DMlncRNA Riskscore	High/Low	1.013	1.009–1.017	<0.001	1.009	1.004–1.013	<0.001
Age		1.060	1.044–1.075	<0.001	1.057	1.040–1.074	<0.001
Gender	Female/Male	1.032	0.720–1.479	0.864	—	—	-—
Grade	G2/G3	3.299	2.218–4.907	<0.001	1.920	1.224–3.011	0.004
Diagnoses	Astrocytoma, anaplastic/Astrocytoma, NOS/Mixed glioma/Oligodendroglioma, anaplastic/Oligodendroglioma, NOS	0.751	0.662–0.851	<0.001	0.798	0.694–0.918	0.002
Type	Primary/Recurrent	1.750	0.852–3.592	0.127	—	—	—
IDH1 R132status	Mutation/Wild	2.898	2.000–4.200	<0.001	2.303	1.471–3.605	<0.001
EGFR status	Mutation/Wild	0.312	0.183–0.534	<0.001	0.957	0.481–1.904	0.901
ATRX status	Mutation/Wild	1.359	0.938–1.970	0.105	—	—	—
TP53 status	Mutation/Wild	1.275	0.891–1.825	0.184	—	—	—
PTEN status	Mutation/Wild	0.511	0.280–0.933	0.029	1.430	0.676–3.025	0.349

**FIGURE 6:**
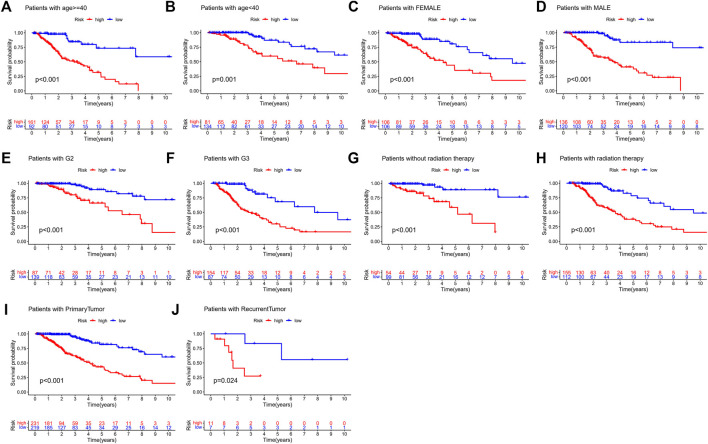
**(A–J)** Overall survival patterns of patients grouped based on tumour grade, sex, age radiation therapy and tumour type (primary/recurrent) in low- and high-risk patients in TCGA cohort.

In addition, we performed a stratified analysis of the DMlncRNA risk score to examine whether its predictive value was dependent on tumour type, tumour grade, radiotherapy, sex and age. Patients in TCGA cohort were divided into the following two groups based on the median age of patients (40 years): a group with patients aged ≥40 years (*n* = 253) and a group with patients aged <40 years (*n* = 215). Based on the DMlncRNA risk score, patients in the two groups were further divided into the low- and high-risk groups. OS was significantly different between the low- and high-risk groups in the group with patients aged <40 years (*p* < 0.001) (Figure 6A) and in the group with patients aged ≥40 years (*p* < 0.05) (Figure 6B). Furthermore, patients were grouped based on sex, and 212 patients were women and 256 patients were men in TCGA cohort. These patients were further divided into the low- and high-risk groups based on their risk scores. OS was significantly higher in the low-risk group than in the high-risk group (*p* < 0.001, Figures 6C,D). Furthermore, patients were grouped based on tumour grade, and 241 patients were included in the Grade III group and 226 patients were included in the Grade II group in TCGA cohort. Patients in the Grade II group were divided into the low- and high-risk groups based on the DMlncRNA risk scores, and OS was significantly different in both groups (*p* < 0.001) (Figure 6E). Similarly, the DMlncRNA risk scores were used to divide patients in the Grade III group into the low- and high-risk groups, and OS was significantly different in both groups (*p* < 0.001) (Figure 6F). Finally, stratification analyses were performed based on tumour type (primary and recurrent) and whether patients received radiotherapy. The findings indicated that OS was significantly lower in the high-risk group than in the low-risk group, irrespective of whether patients received radiotherapy and tumour type (*p* < 0.05) (Figures 6G–J). These findings suggested that the DMlncRNA risk score was an independent factor for predicting the OS of patients with LGG.

### Verification of the DNA Methylation Regulator-Related lncRNA Signature in Three External Independent LGG Datasets

**FIGURE 7:**
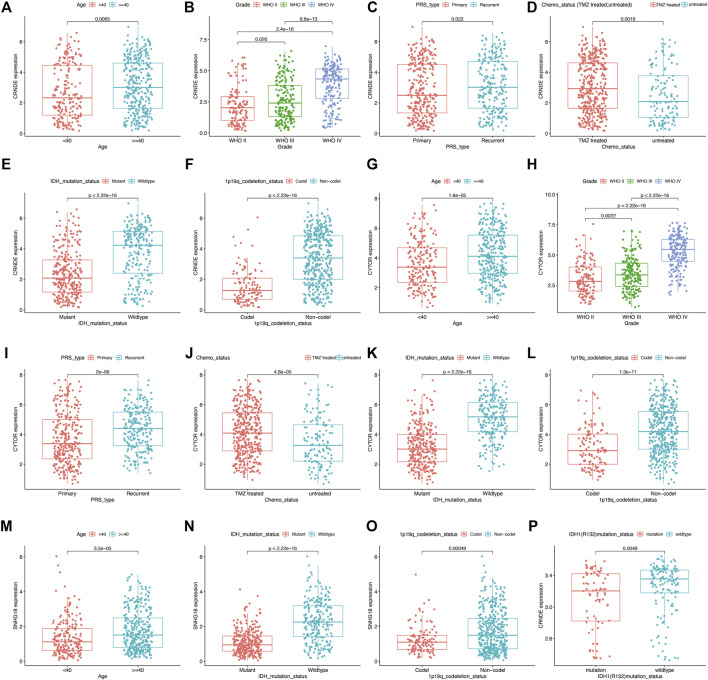
Prognostic value of DNA methylation-related lncRNAs (DMlncRNAs) evaluated using the CGGA mRNA-seq-693 and GSE16011 datasets as external independent cohorts. **(A–L)** Box plots showing the expression levels of CRNDE and CYTOR in patients grouped based on 1p19q codeletion status, tumour grade, age (<40 and ≥40 years), PRS type, chemotherapy status and IDH1 mutation status in the CGGA mRNA-seq-693 cohort (RNA-seq data). **(M–O)** Box graphs showing the expression level of SNHG18 in patients grouped based on IDH1 mutation status, 1p19q codeletion status and age in the CGGA mRNA-seq-693 cohort. **(P)** Box plots showing the expression level of CRNDE in patients grouped based on IDH1 (R132) mutation status in the GSE16011 cohort.

The CGGA mRNA-seq-693 (*n* = ) and CGGA mRNA-seq-325 (*n* = ) datasets were used to validate the prognostic performance of DMlncRNAs. First, we investigated the relationship between the lncRNAs CRNDE, CYTOR and SNHG18 and DNA methylation in the CGGA mRNA-seq-693 cohort. The results indicated that the expression levels CRNDE and CYTOR were significantly correlated (*p* < 0.05) with tumour grade, age (<40 and ≥40 years), PRS type, 1p19q chromosome codeletion, IDH mutation status and chemotherapy status (Figures 7A–L). Furthermore, SNHG18 was significantly correlated (*p* < 0.05) with IDH mutation status and age (<40 and ≥40 years), and SNHG18 expression levels were significantly different in the 1p19q chromosome codeletion subgroups (Figures 7M–O). In addition, CRNDE exhibited a good correlation (*p* < 0.05) with IDH1 (R132) mutation status (Figure 7P) in the GSE16011 dataset.

Similar results were observed in the CGGA mRNA-seq-325 dataset for the lncRNAs CRNDE, CYTOR and SNHG18. the expression levels of these lncRNAs were strongly correlated with chromosome 1p19q codeletion, IDH mutation status, tumour grade, chemotherapy status and age (<40 and ≥40 years) (*p* < 0.05) (Supplementary Figures 2A–O).

### Five DNA Methylation Regulator-Related lncRNAs Were Implicated in Immune Infiltration

**FIGURE 8:**
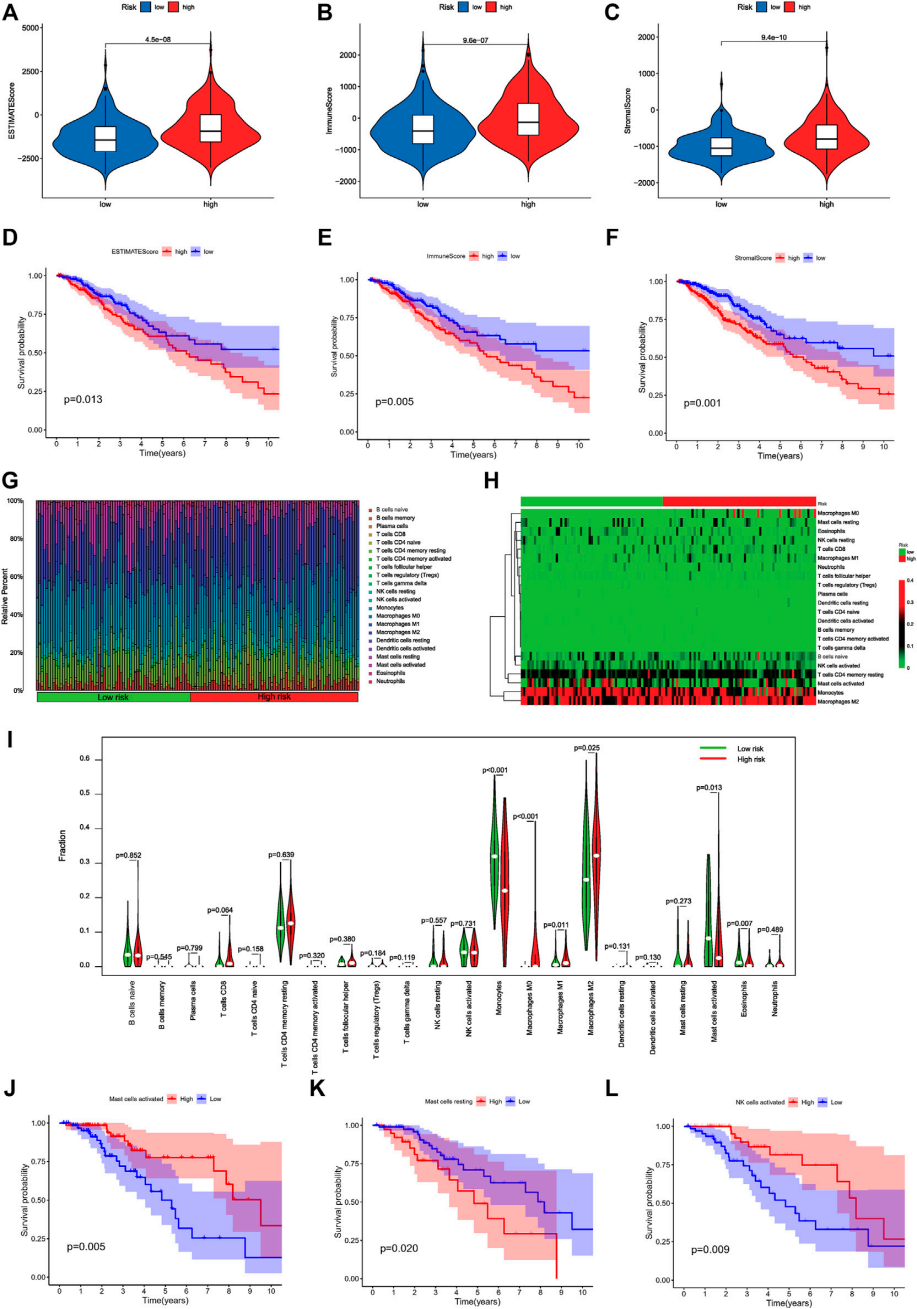
Correlation of risk scores with immune cell infiltration and overall survival in LGGs. **(A–C)** Relationship among the ESTIMATE, immune and stromal scores in the high- and low-risk groups. **(D)** Prognostic value of ESTIMATE scores as determined *via* Kaplan–Meier survival analysis. **(E)** Prognostic significance of immune scores as determined *via* Kaplan–Meier survival analysis. **(F)** Prognostic significance of stromal scores as determined *via* Kaplan–Meier survival analysis. **(G)** Distribution diagram of 22 types of TICs in different risk patients. **(H)** The Heatmap of expression of 22 types of TICs in different risk patients. The deepening of the red color indicates an increased level of expression. **(I)** Differentiation ratio of 22 immune cell types between the low- and high-DMlncRNA-expression groups. **(J–L)** Kaplan–Meier survival analysis for activated NK cells, resting mast cells and activated mast cells in the low- and high-risk groups.

We evaluated immune scores and immune cell infiltration levels in the high- and low-risk groups to examine the role of DMlncRNAs in immune cell infiltration in LGGs. The ESTIMATE (Figure 8A, *p* = 4.5e-08), immune (Figure 8B, *p* = 9.6e-07) and stromal (Figure 8C, *p* = 9.4e-10) scores of the high-risk group were higher than those of the low-risk group. The ESTIMATE, immune and stromal scores of patients with LGGs were significantly correlated with prognosis (Figures 8D–F) (*p* < 0.05). The survival rate of the high-score group was lower than that of the low-score group, implying that TME features are highly associated with the onset of LGG progression. Furthermore, the association between the expression of DMlncRNAs and immune components was determined *via* the CIBERSORT algorithm using 22 types of immune cell profiles in the low- and high-risk groups. A total of 149 tissues were found to be eligible for the analysis of CIBERSORT (*p* < 0.05). In addition, the proportion of tumour-infiltrating immune subtypes was evaluated, and heat maps were generated (Figures 8G,H). Interestingly, the infiltration of M2-type macrophages and monocytes was mainly in all LGG samples. Among the high-risk patients had a higher infiltration of M0-type macrophages. In contrast, low-risk patients seemed to have a higher infiltration of naive B cells. Correlation and differential analyses revealed six TIC types that were strongly correlated with the expression of DMlncRNAs (Figure 8I) (*p* < 0.05). Finally, immune cells were divided into the high- and low-expression groups based on the median. The results revealed that OS was better in the high-expression group with activated mast cells and activated NK cells than in the low-expression group (Figures 8J,L) (*p* < 0.05), whereas OS was better in the low-expression group with resting mast cells than in the high-expression group (Figure 8K) (*p* < 0.05). These results indicated that the expression of DMlncRNAs significantly affected the immune activity in TME.

### GO and KEGG Enrichment Analyses

**FIGURE 9:**
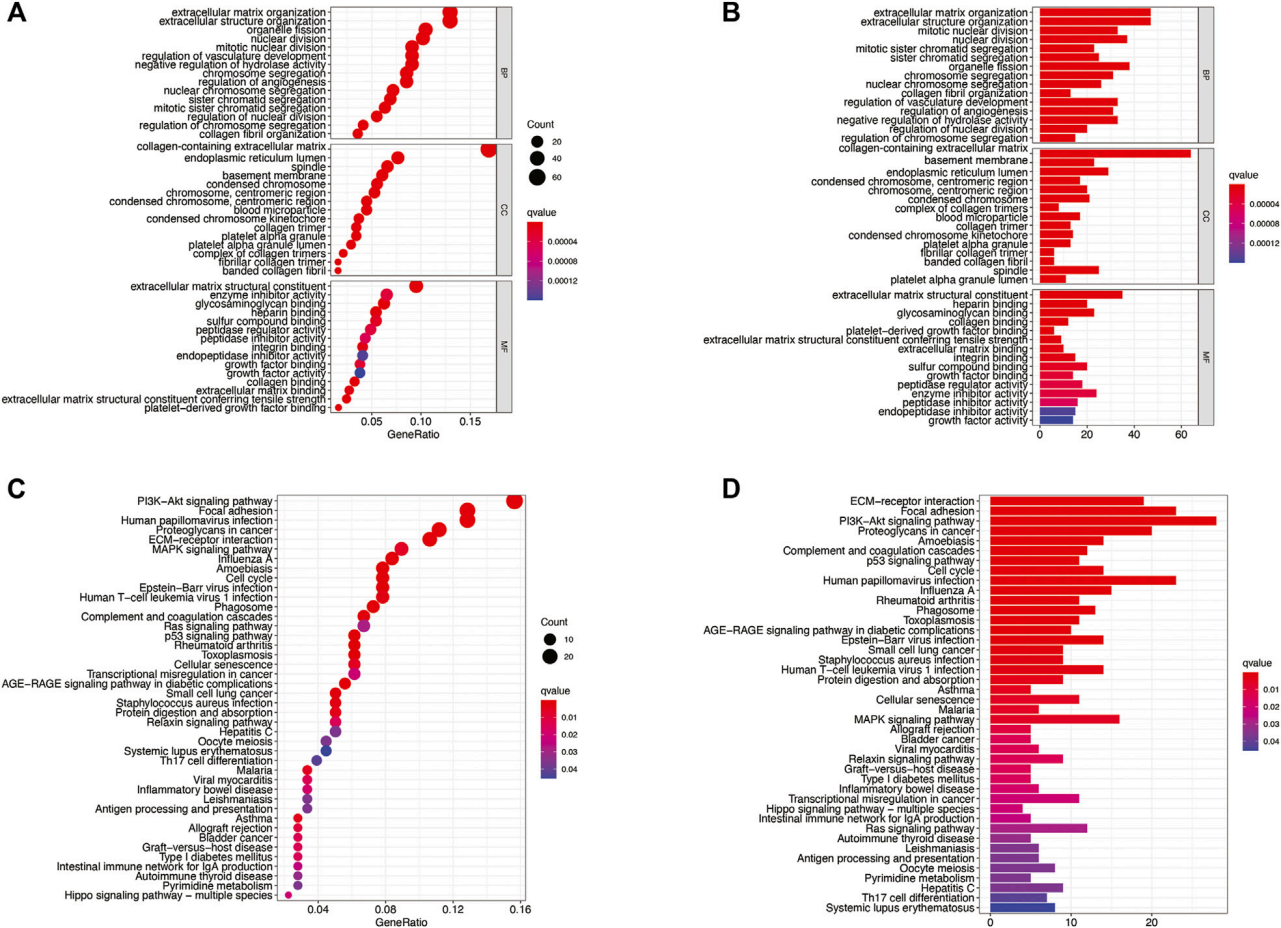
**(A–D)** KEGG and GO enrichment analyses, with significant enrichment indicated by *p* and *q* < 0.05.

To further evaluate the biological role of DMlncRNAs in LGGs, we screened for differentially expressed mRNAs in the low- and high-risk groups using the most enriched KEGG pathways and GO terms related to molecular functions (MFs), cellular components (CCs) and biological processes (BPs). The GO terms were mainly associated with extracellular matrix organisation, structural organisation, structural components of the extracellular matrix, sister chromatid separation and chromosome segregation involved in DNA methylation associated with genomic instability (Figures 9A,B). In addition, KEGG analysis revealed that DMlncRNAs were mainly enriched in the Hippo signalling pathway, MAPK signalling pathway, PI3K–Akt signalling axis, p53 signalling pathway, Ras signalling pathway, transcriptional dysregulation in cancer, antigen processing and presentation and autoimmune thyroid disease (Figures 9C,D). These signalling pathways are associated with core biological oncogenic processes, most of which involve regulation of the oncogenic activation pathways and immune checkpoint expression. Therefore, the effects of these pathways on DNA methylation modifications during immunotherapy should be investigated further.

### Construction of ceRNA Network

For exploring the potential regulatory relationship of lncRNAs in risk signature, we combined the results of multiple databases to construct a ceRNA network. Firstly, We used starbase online tool to explore the lncRNA-miRNA axis; however, unfortunately, only CRNDE and CYTOR had prediction results for downstream miRNAs. Next, we explored target-mRNAs of miRNAs in three databases (miRDB, miRtarBase, and TargetScan). The above target-mRNAs were overlapped with differentially expressed genes to obtain the final target genes that may be involved in genomic instability. It's worth noting a total of 1,040 differentially expressed mRNAs were identified in the GS- and GU-like groups (log FC filter| > 0.585, FDR-adjusted *p*-value < 0.05; Wilcoxon test). Finally, we constructed a ceRNA network (316 edges) based on 2 lncRNAs, 112 miRNAs, and 58 mRNAs (Supplementary Figure S3). Interestingly, CRDNE may be closely associated with a higher number of genomic instability-associated mRNAs. In microRNAs, mir-29 family (mir-29a-3p, mir-29b-3p, and mir-29c-3p) had the largest number of edges, which may indicate the important regulatory in ceRNA network.

### Validation of the Expression of Four lncRNAs

**FIGURE 10:**
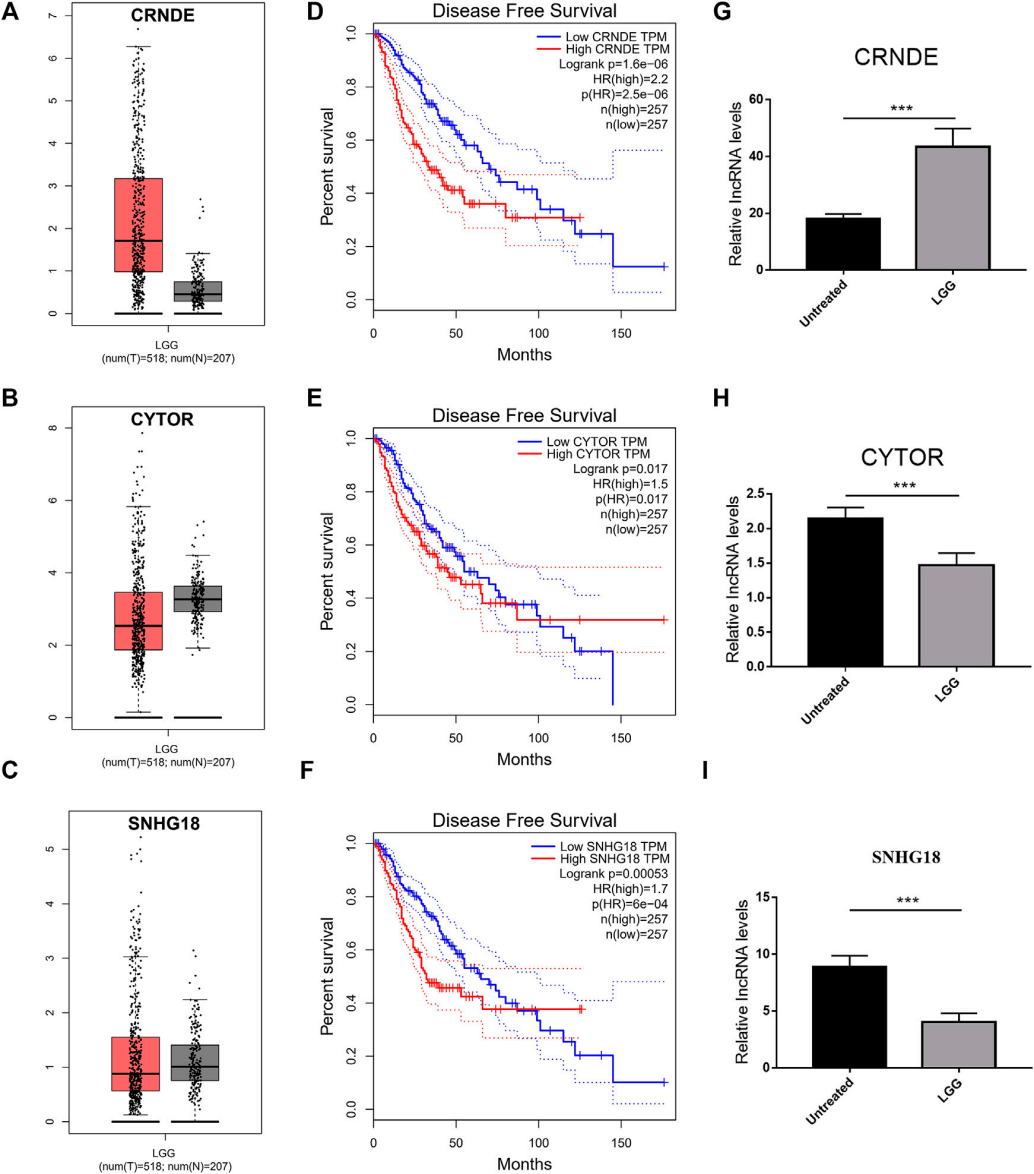
Validation of the expression of DMlncRNAs. **(A–C)** Comparison of the expression profiles of three lncRNAs (CRNDE, CYTOR and SNHG18) between TCGA (518 LGG samples) and GTEx (207 normal brain samples) cohorts *via* GEPIA. **(D–F)** Disease-free survival evaluated based on three lncRNAs *via* Kaplan-Meier survival curves. **(G–I)** Bar plots demonstrating the expression of three lncRNAs in LGG and normal brain samples evaluated *via* qRT-PCR (***p < 0.001).

To verify the expression of DMlncRNAs, three lncRNAs were first analysed using the GEPIA and GTEx databases. The findings indicated that the expression level of lncRNA CRNDE was significantly higher in LGG samples than in normal brain specimens (*p* < 0.05; Figure 10A). However, the expression levels of CYTOR and SNHG18 were lower in LGG tissues than in normal brain tissues (Figures 10B,C). Furthermore, survival analysis was performed using GEPIA to examine the association between survival rates and the lncRNAs CRNDE, CYTOR and SNHG18. Low expression levels of DMlncRNAs were significantly (*p* < 0.05, Figures 10D–F) associated with the DFS of patients with LGGs. Finally, to better characterise the expression levels of DMlncRNAs in normal and LGG tissues, eight normal brain tissue samples and eight LGG samples were collected. The expression levels (qRT-PCR) of CRNDE and MPPED2-AS1 were significantly higher in LGG samples than in normal brain tissue samples (*p* < 0.05, Figures 10G–I, Supplementary Figure S4). However, the expression levels of SNHG18 and CYTOR were higher in normal brain samples than in LGG samples (*p* < 0.05), which was consistent with the results of GEPIA.

### Potential Candidate Drug for LGGs

**FIGURE 11:**
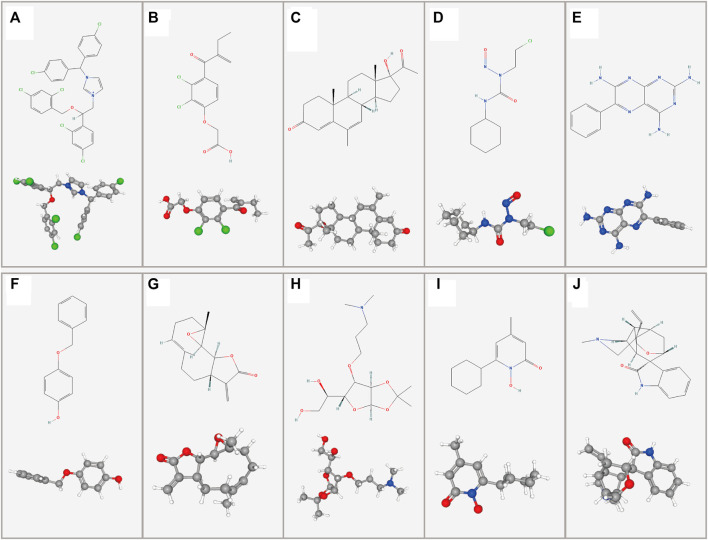
Two- and three-dimensional conformers of the selected compounds obtained *via* CMap analysis. **(A)** Calmidazolium, **(B)** Etacrynic acid, **(C)** Megestrol, **(D)** Lomustine, **(E)** Triamterene, **(F)** Monobenzone, **(G)** Parthenolide, **(H)** Amiprilose, **(I)** Ciclopirox, **(J)** Gelsemine.

Potential drug compounds significantly associated with the differentially expressed DMlncRNAs were identified using the cMap database. We found that 10 small molecule drugs were negatively associated with LGG, such as calmidazolium (specificity = 0.123), etacrynic acid (specificity = 0.006), monobenzone (specificity = 0.0405), parthenolide amiprilose (specificity = 0.1724), ciclopirox (specificity = 0.0952) and gelsemine (specificity = 0.0409), which may inhibit the development of LGG (Figure 11, Supplementary Table S2). It is worth mentioning that we found that calmidazolium could cross the blood-brain barrier (Lee and Hait, 1985).

## Discussion

DNA methylation involves the addition of a methyl group to a DNA base through a covalent linkage. It is caused by agents that alkylate DNA and damage it or a highly regulated mechanism that results in DNA epigenetic modifications. Methylation that results in DNA damage is associated with specific modifications [primarily at position 5 of cytosine (5mC)] and production of N3-methylcytosine (m3C) and changes in N1-methyladenine (m1A) (J Dabrowski and Wojtas, 2019) and plays a key role in post-transcriptional gene expression modulation. Aberrant DNA methylation is involved in cancer progression *via* modulation of several cellular processes, such as cell proliferation, immune response and genomic instability (Veland et al., 2019). It has been shown that extensive hypomethylation in cells occurs concomitantly with CpG island hypermethylation as the most characteristic DNA methylation pattern in glioma cells (Zang et al., 2018).

DNA methylation may regulate gene expression and genomic instability in LGGs in different ways as follows: 1) Gene promoter hypermethylation prevents transcription factors (TFs) from binding to DNA sequences and may lead to silencing of gene expression once the promoter is demethylated (Tough et al., 2020). 2) When the gene promoter is hypermethylated, it can bind to a transcriptional repressor (REP), thereby repressing gene expression. The repressor is released if the promoter is demethylated, resulting in gene expression (J Dabrowski and Wojtas, 2019). 3) Binding of twoTFs to promoters stimulates gene expression, with one TF mainly binding to methyl groups and the other TF preferentially binding to unmethylated DNA (Suzuki et al., 2017). 4) Under normal conditions, 5mC is oxidised to 5hmC by TET proteins, and other forms such as 5caC and 5fC are also produced (Brandt et al., 2019). Inhibition of TET by 2-hydroxyglutarate (2HG) results in IDH1/2 mutations; therefore, TET cannot catalyse the formation of 5hmC through demethylation, resulting in DNA hypermethylation (Ludwig et al., 2016). 5) DNA methylation is associated with the opening of chromatin because chaperones of chromatins are more sensitive to DNA expression and activation or inhibition of histones. ATRX proteins can be ligated to methylated DNA, leading to the formation of heterochromatin, thus inhibiting access to TFs and preventing gene transcription and expression (Teng et al., 2021). Hypermethylation of CTCF binding sites dissociates CTCF, leading to changes in chromatin conformation, i.e., genomic instability, which affects gene transcription (Kawashima et al., 2012).

Several studies have explored lncRNA signatures to determine the OS of patients with cancer. However, studies investigating the role of DMlncRNAs in predicting patient survival and the genomic instability and immune microenvironment of malignant tumours, including LGGs, are limited. Studies should evaluate DNA methylation profiles of individual tumours for effective prognosis (in LGGs) owing to the heterogeneity of DNA methylation modifications. In the present study, we assessed the prognostic value and identified molecular drug targets of DMlncRNAs. In addition, we investigated the association between immune infiltration and DMlncRNAs in LGGs.

In our study, a prognostic signature for LGG was established using the five prognostic DMlncRNAs, which has not been previously reported. The 5-DMlncRNA signature exhibited good discriminatory properties for predicting the prognosis of patients with LGGs. KEGG and GO enrichment analyses demonstrated that chromosome segregation, Hippo signalling pathway, MAPK signalling pathway, PI3K–Akt signalling pathway, p53 signalling pathway and Ras signalling pathway were associated with the low- and high-risk groups. DNA methylation in mitotic repeat regions is essential for genomic stability (e.g., chromosome segregation in mitosis) and has the potential to inhibit transposable factor expression, thereby affecting genomic stability (Yamane et al., 2011). The P53 signalling pathway is involved in several cell processes, such as enhanced DNA repair, differentiation, genomic instability and cell death after cellular stress, inhibition of cell cycle progression, inhibition of growth and apoptosis by modulating gene expression (Nakanishi et al., 2015), and is regulated *via* post-translational methylation, phosphorylation, acetylation, ubiquitination and other modifications (Cheng et al., 2018). The PI3K–Akt signalling pathway plays a critical role in the progression and malignant proliferation of glioma cells. For example, lncRNA FOXD2-AS1 modulates the PI3K/AKT signalling pathway and miR-185-5P/HMGA2 axis to promote the progression of glioma (Ni et al., 2019); miR-3116 increases the sensitivity of glioma cells to temozolomide (TMZ) by inhibiting FGFR1 and inactivating the PI3K/AKT pathway (Kong et al., 2020) and exosome-mediated MIF by regulating the glioma TIMP3/PI3K/AKT axis (Li M. et al., 2017). In addition, the MAPK signalling pathway is present in most cells and play a crucial role in transducing signals from extracellular stimuli to the cell and its nucleus and eliciting cell biological responses (e.g., cell proliferation, differentiation, genomic instability and apoptosis) (Cao et al., 2020b). The Ras signalling pathway has been extensively investigated as a potential therapeutic target regulating cell apoptosis in LGGs and genomic instability is an essential hallmark of LGGs (da Fonseca et al., 2008).

The lncRNA CRNDE is located on chromosome 16q12.2 in humans and was initially reported to be highly expressed in colorectal cancer, functioning as a biomarker, and was subsequently found to have the most upregulated expression in glioma (Kiang et al., 2017). CRNDE is closely related to tumour grade and tumour cell growth and migration and can affect gene expression by regulating epigenetic modifications, such as histone methylation. Recent studies have shown that CRNDE regulates autophagy and ABCG2 expression through the PI3K/Akt/mTOR pathway as a potential biomarker for predicting the treatment response to TMZ and modulating TMZ sensitivity in GBM(Zhao et al., 2021). CRNDE promotes glioma malignancy by acting as ceRNA and blocking the downregulation of Bcl-2 and Wnt2 mediated by miR-136-5ps (Li D.-X. et al., 2017).

In addition, CRNDE is involved in the initiation and tumorigenesis of several cancers. For example, it promotes atg4b-mediated autophagy and attenuates sorafenib sensitivity in hepatocellular carcinoma cells (Chen et al., 2021); it regulates eIF4A3/MUC1/EGFR signalling and modulates the response of EGFR-mutant lung cancer to EGFR tyrosine kinase inhibitor resistance (Takahashi et al., 2021). In addition, CRNDE expression is modulated *via* DNA methylation, and CRNDE exhibits protective effects on CLL by preventing CLL progression through the miR-28/NDRG2 axis (Ni et al., 2021). The lncRNA CYTOR has been reported to regulate L-OHP resistance and promote EMT in colon cancer cells *via* miR-378a-5p/SERPINE1(Yang et al., 2021). In addition, it reduces radiosensitivity in non-small cell lung cancer by inhibiting miRNA-206 expression and activating prothymosin α(Jiang et al., 2021). The lncRNA SNHG18 has been identified as a novel prognostic biomarker for tumours in previous studies, and high SNHG18 expression is correlated with a poor prognosis. For example, SNHG18 knockdown suppressed metastasis and invasion of gliomas (Huang et al., 2021). Furthermore, mkl1-induced SNHG18 regulates the metastasis and growth of non-small cell lung cancer by modulating the miR-211-5p/BRD4 pathway (Fan et al., 2021). However, the biological functions of MPPED2-AS1 and AC010273.2 have not been comprehensively elucidated. In addition, mechanistically, some lncRNAs with specific miRNA target sites are capable of regulating gene expression *via* acting as ceRNAs (Thomson and Dinger, 2016). We constructed the ceRNA network to show two lncRNAs in risk signature with their binding miRNAs and target genes. Especially, mir-29 family (mir-29a-3p, mir-29b-3p, and mir-29c-3p) has the largest number of edges, which may indicate the important regulatory in ceRNA network. There is no doubt that research has demonstrated the importance of mir-29 family. miR-29b potentiates TMZ sensitivity against GBM cells by inducing autophagy and the combined use of miR-29 mimic and TMZ might represent a potential therapeutic strategy for GBM patients (Xu et al., 2021). Interestingly, the predictive value of serum miR-29 family in high-graded glioma detection was sufficient (AUC = 0.81) (Wu et al., 2015).

In this study, validation analyses performed using several bioinformatic databases and cohorts and the literature review suggest that DMlncRNAs can predict the survival rate of patients with cancer and serve as indicators of cancer.

In immune analysis, the immune, stromal and ESTIMATE scores were higher in high-risk patients than in low-risk patients. In addition, the prognosis of patients in the high-expression group with activated mast cells and activated NK cells was better than that of patients in the low-expression group, whereas the survival of patients in the low-expression group with resting mast cells was better than that of patients in the high-expression group. Finally, the cMap webserver was used to screen for candidate drugs significantly associated with differentially expressed DMlncRNAs in LGGs. Unfortunately, there are no randomized controlled studies of the application of candidate drugs in clinical work. Hence, this is a further reminder of the therapeutic potential of small molecule drugs for different risk patients.

Although the findings of the present study offer insights into the relationship between DMlncRNAs and LGG prognosis, some limitations need to be addressed and investigated further. First, the mechanisms of action of DMlncRNAs in affecting tumour immunity remain unclear. The specific mechanisms should be elucidated further. Second, the role of DMlncRNAs was examined using bioinformatic techniques and qRT-PCR assays, and data were verified using only three external independent datasets. Hence, Larger cohorts will be needed for validation in the future.

Therefore, this study provides a critical prognostic strategy, offers novel insights into the function of lncRNAs in DNA methylation and reveals the possible pathways of modulation of tumour progression *via* DNA methylation.

## Data Availability

The datasets presented in this study can be found in online repositories. The names of the repository/repositories and accession number(s) can be found in the article/Supplementary Material.
