# Supplementary figures and images for: Modification Patterns of DNA Methylation-Related lncRNAs Regulating Genomic Instability for Improving the Clinical Outcomes and Tumour Microenvironment Characterisation of Lower-Grade Gliomas
Source: Front Mol Biosci. 2022 Mar 10;9:844973. doi: 10.3389/fmolb.2022.844973 (PMC8960387; doi:10.3389/fmolb.2022.844973)

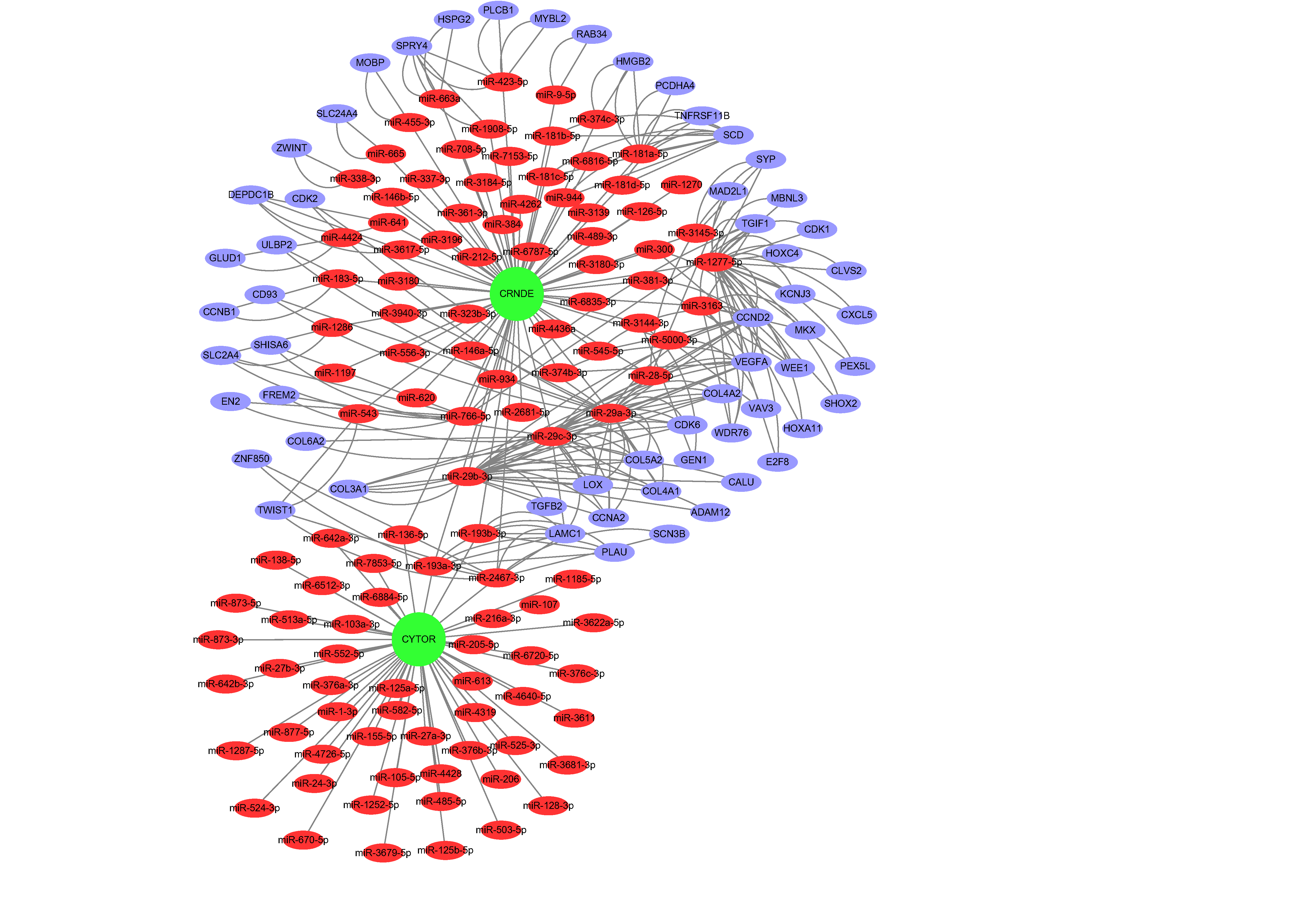

Supplement: Supplementary file 1 [file Image3.TIFF]

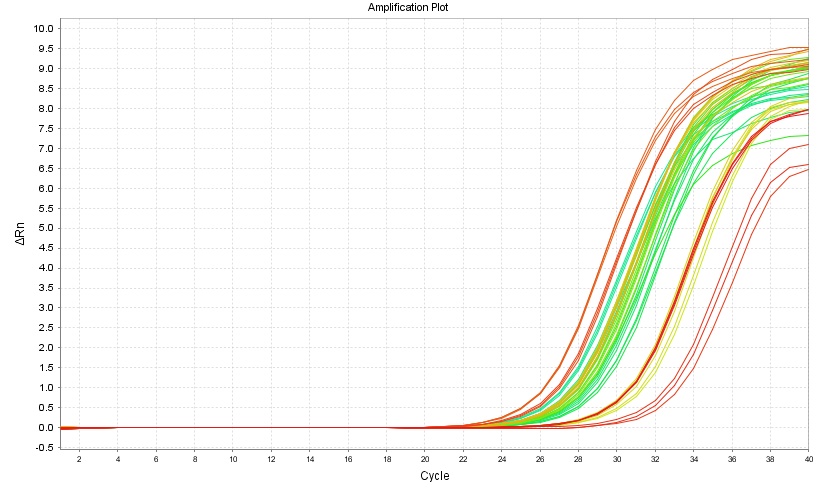

Supplement: Supplementary file 3 [file Image9.JPEG]

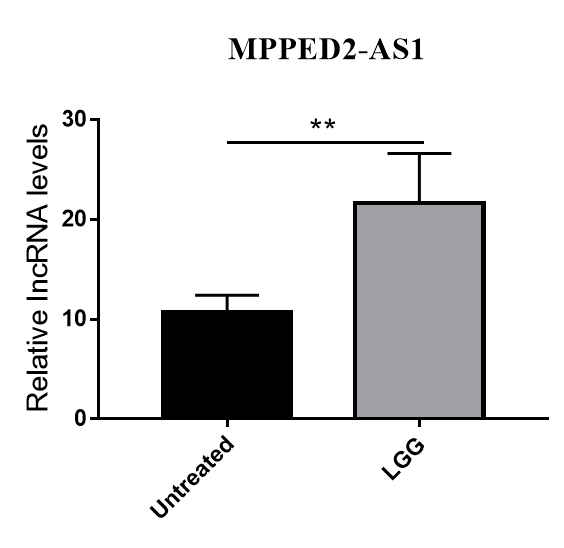

Supplement: Supplementary file 5 [file Image4.TIF]

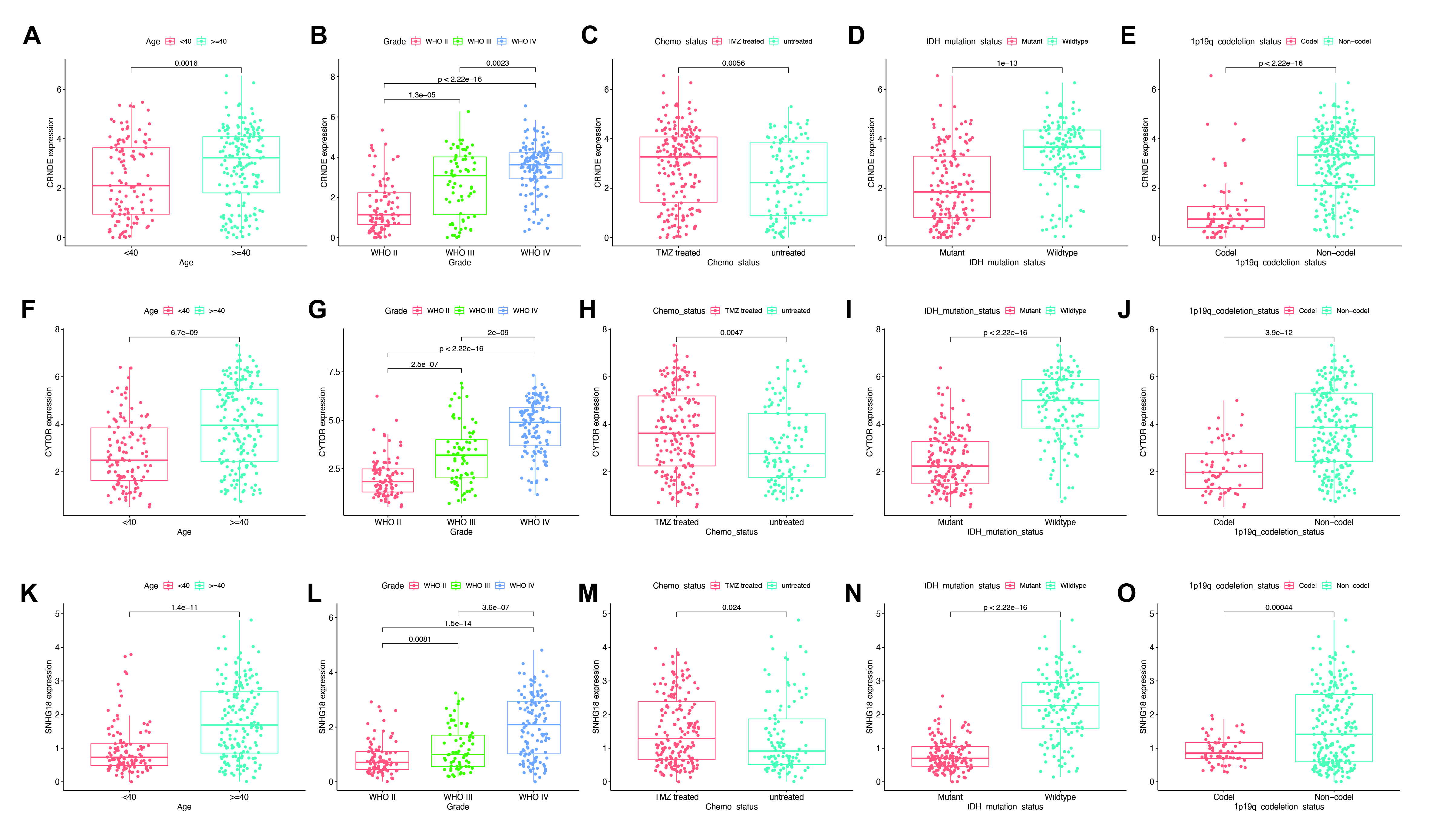

Supplement: Supplementary file 6 [file Image2.TIF]

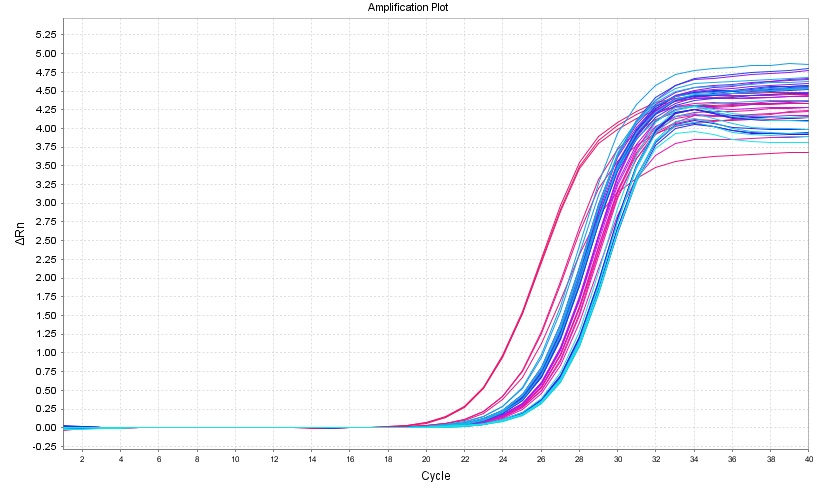

Supplement: Supplementary file 7 [file Image7.JPEG]

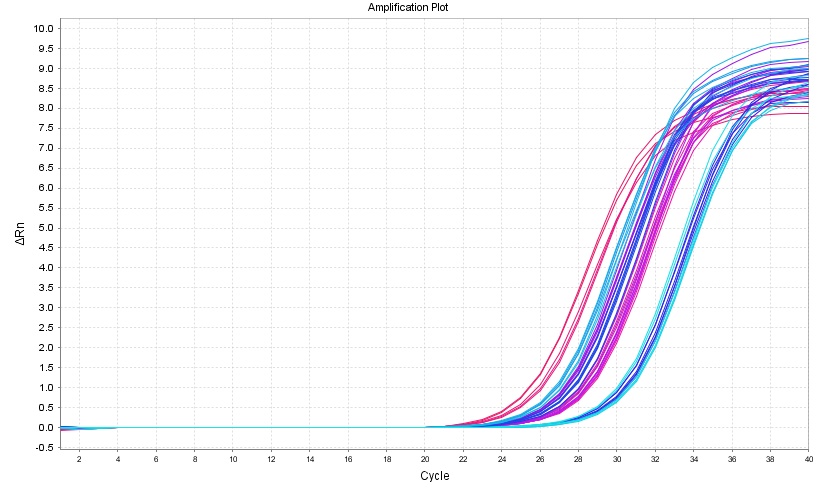

Supplement: Supplementary file 8 [file Image5.JPEG]

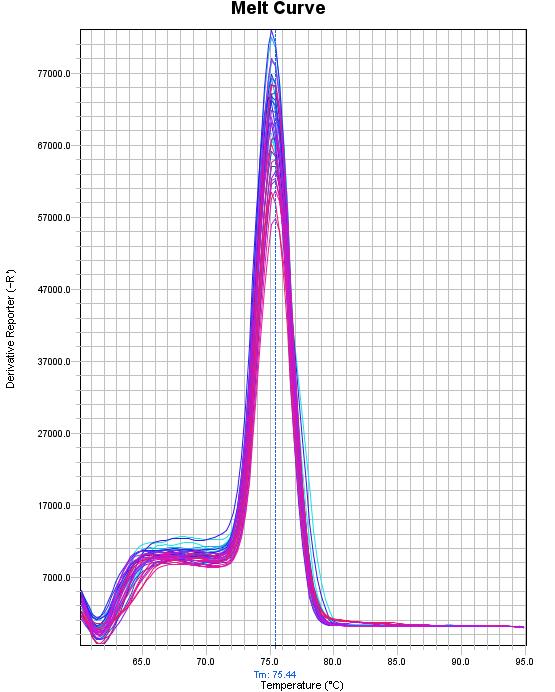

Supplement: Supplementary file 9 [file Image10.JPEG]

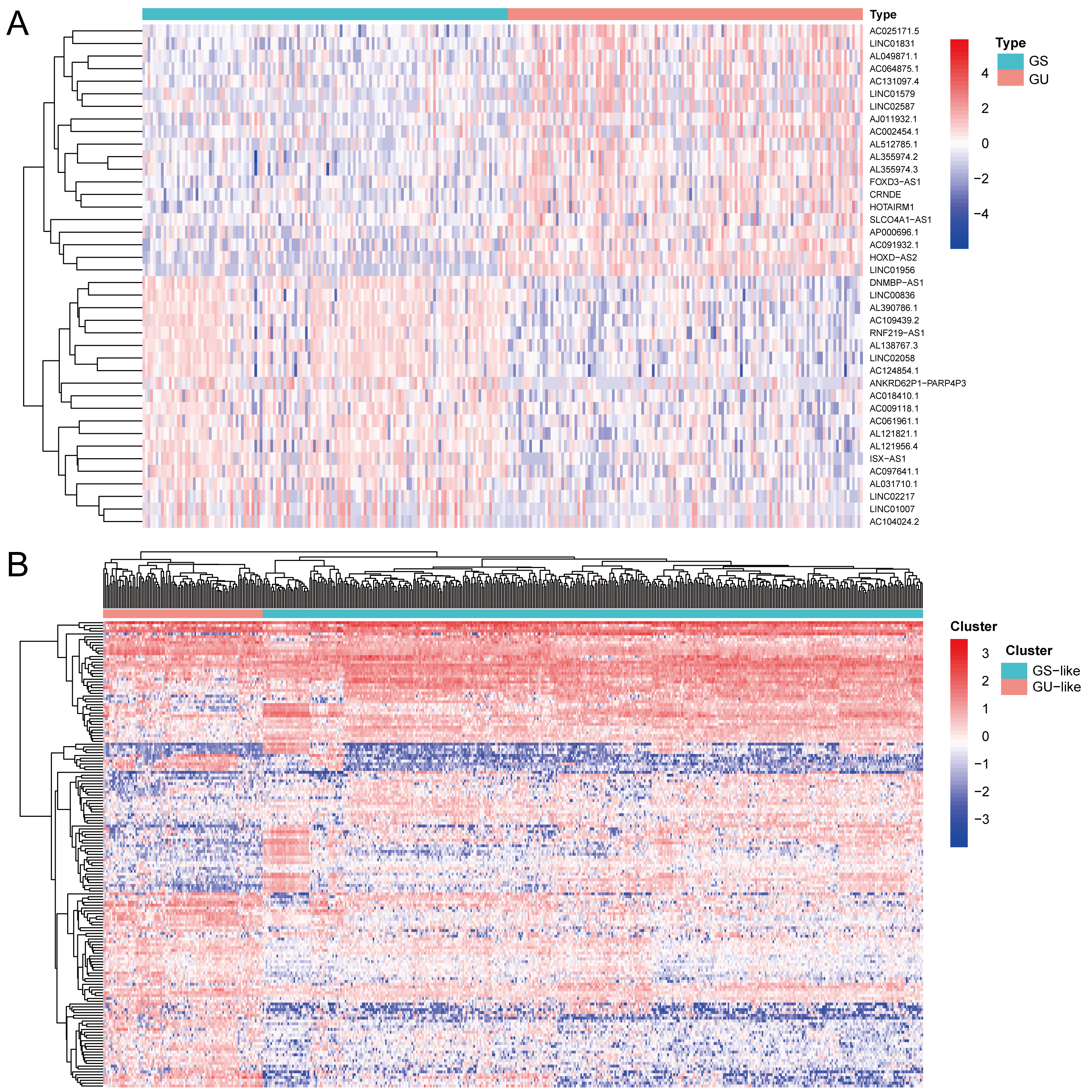

Supplement: Supplementary file 10 [file Image1.TIF]

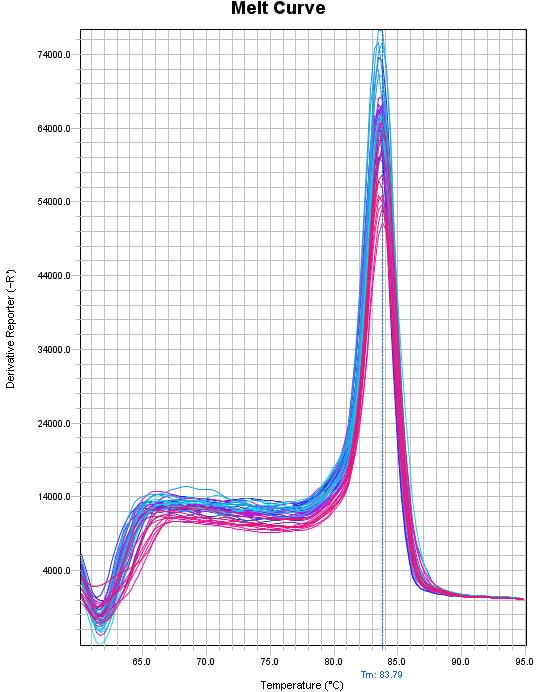

Supplement: Supplementary file 11 [file Image14.JPEG]

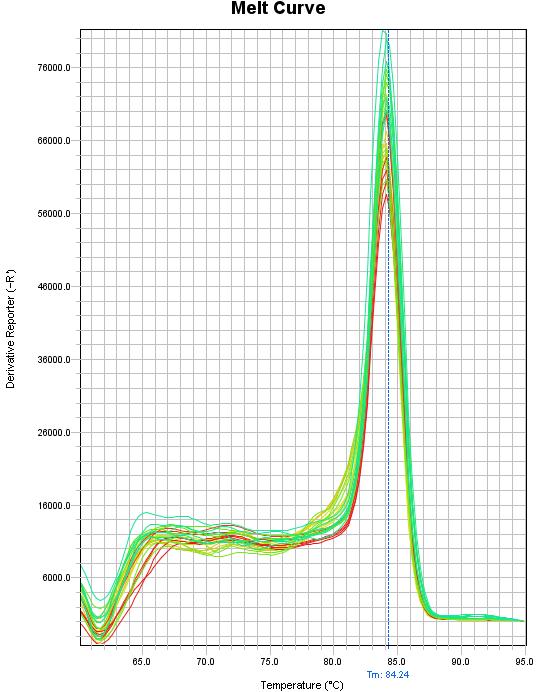

Supplement: Supplementary file 12 [file Image12.JPEG]

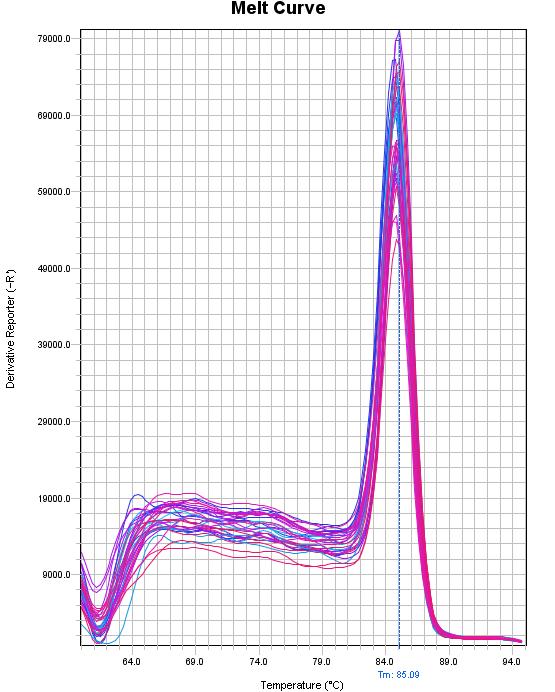

Supplement: Supplementary file 13 [file Image11.JPEG]

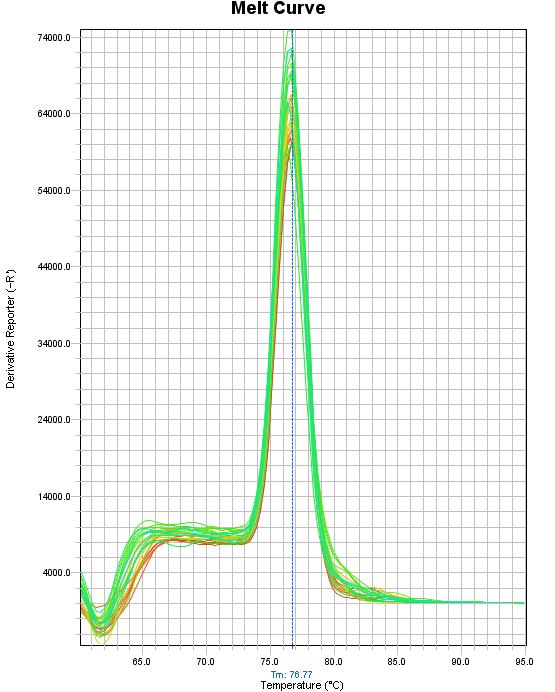

Supplement: Supplementary file 18 [file Image13.JPEG]

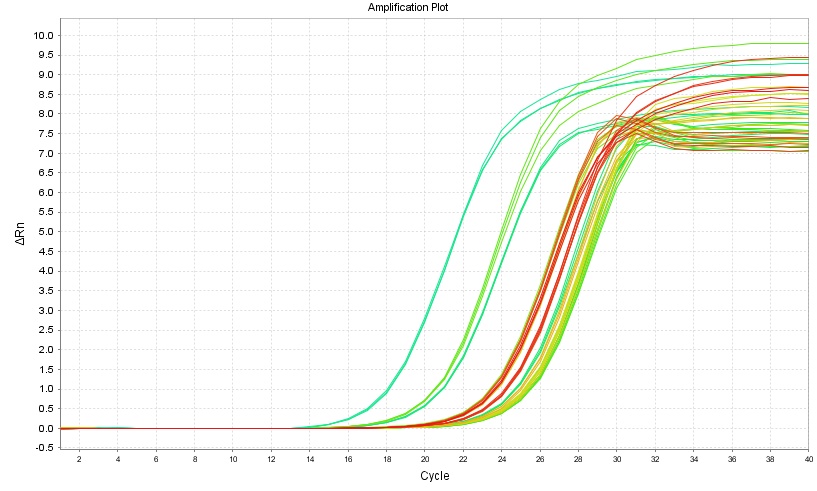

Supplement: Supplementary file 20 [file Image8.JPEG]

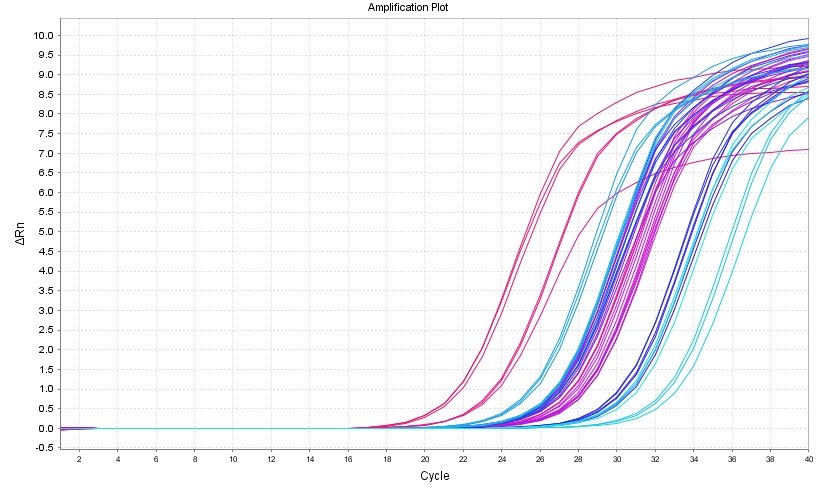

Supplement: Supplementary file 25 [file Image6.JPEG]
