# Supplementary material for: Modification Patterns of DNA Methylation-Related lncRNAs Regulating Genomic Instability for Improving the Clinical Outcomes and Tumour Microenvironment Characterisation of Lower-Grade Gliomas
Source: Front Mol Biosci. 2022 Mar 10;9:844973. doi: 10.3389/fmolb.2022.844973 (PMC8960387; doi:10.3389/fmolb.2022.844973)
Supplement: Supplementary file 15 [file Table5.DOCX]

**Table S2.** Connectivity map analysis results.

| CMap name | Mean connective score | n | Enrichment | P value | Specificity | Percent non-null |
| --- | --- | --- | --- | --- | --- | --- |
| Calmidazolium | -0.511 | 2 | -0.854 | 0.04269 | 0.123 | 100 |
| Etacrynic acid | -0.5 | 3 | -0.845 | 0.00741 | 0.006 | 100 |
| Megestrol | -0.35 | 4 | -0.76 | 0.00684 | 0 | 75 |
| Lomustine | -0.39 | 4 | -0.723 | 0.012 | 0.0845 | 50 |
| Triamterene | -0.44 | 5 | -0.704 | 0.00495 | 0.0184 | 80 |
| Monobenzone | -0.347 | 4 | -0.692 | 0.0193 | 0.0405 | 50 |
| Parthenolide | -0.421 | 4 | -0.676 | 0.02445 | 0.1724 | 75 |
| Amiprilose | -0.315 | 4 | -0.676 | 0.02459 | 0.0177 | 75 |
| Ciclopirox | -0.24 | 4 | -0.672 | 0.02594 | 0.0952 | 50 |
| Gelsemine | -0.463 | 4 | -0.671 | 0.0262 | 0.0409 | 75 |
