# Supplementary material for: Modification Patterns of DNA Methylation-Related lncRNAs Regulating Genomic Instability for Improving the Clinical Outcomes and Tumour Microenvironment Characterisation of Lower-Grade Gliomas
Source: Front Mol Biosci. 2022 Mar 10;9:844973. doi: 10.3389/fmolb.2022.844973 (PMC8960387; doi:10.3389/fmolb.2022.844973)
Supplement: Supplementary file 17 [file Table4.DOCX]

**Table S1.** The differentially expressed genomic unstable and genomic stable of DNA Methylation-related lncRNAs in Low-grade glioma patients.

| lncRNA | conMean | treatMean | logFC | pValue | FDR |
| --- | --- | --- | --- | --- | --- |
| AL021395.1 | 1.010236993 | 0.539283398 | -0.90557825 | 1.89E-05 | 0.000112396 |
| AL031710.1 | 0.809037222 | 0.347418238 | -1.219532586 | 5.44E-07 | 5.45E-06 |
| FOXD2-AS1 | 0.518053255 | 0.795497267 | 0.618756562 | 1.89E-12 | 1.22E-10 |
| AC098851.1 | 0.361879554 | 0.650238768 | 0.845459977 | 0.000289495 | 0.001168861 |
| AL023803.1 | 0.634038863 | 1.01535675 | 0.679343536 | 4.14E-05 | 0.000216043 |
| STXBP5-AS1 | 0.658149096 | 0.383577878 | -0.778894929 | 4.22E-06 | 3.09E-05 |
| AC027130.1 | 1.890527174 | 1.23177651 | -0.618048065 | 1.33E-07 | 1.62E-06 |
| AC055874.1 | 0.382342679 | 0.682235756 | 0.835404121 | 3.27E-05 | 0.000178182 |
| AL022344.1 | 1.003860635 | 0.52831238 | -0.926095873 | 7.23E-12 | 3.59E-10 |
| AL136964.1 | 0.854511434 | 0.46837469 | -0.867436679 | 5.35E-10 | 1.42E-08 |
| AL355974.2 | 3.24717974 | 7.105989012 | 1.129848214 | 1.46E-09 | 3.45E-08 |
| HOTAIRM1 | 0.590971756 | 2.463735804 | 2.059686472 | 3.78E-08 | 5.46E-07 |
| AC015967.1 | 1.286546755 | 0.718903835 | -0.839633183 | 8.02E-13 | 6.20E-11 |
| TMEM72-AS1 | 0.596711937 | 0.341302618 | -0.805983159 | 5.00E-14 | 6.05E-12 |
| AL035461.2 | 1.551970089 | 2.436151311 | 0.65050299 | 1.35E-11 | 6.21E-10 |
| PIK3CD-AS2 | 0.269837474 | 0.539894013 | 1.000585498 | 4.36E-08 | 6.24E-07 |
| Z98884.1 | 0.749586111 | 0.479066889 | -0.645867116 | 3.18E-11 | 1.26E-09 |
| AF131215.7 | 0.338760674 | 0.536583146 | 0.663535338 | 1.04E-05 | 6.55E-05 |
| AC104083.1 | 3.756838787 | 2.497364123 | -0.589113023 | 6.60E-08 | 8.75E-07 |
| LINC02166 | 0.577921462 | 0.894770333 | 0.630643977 | 5.54E-07 | 5.53E-06 |
| AL160270.1 | 0.595004548 | 0.366705607 | -0.69827837 | 9.29E-09 | 1.58E-07 |
| AC023137.1 | 0.701238709 | 0.464539693 | -0.594103764 | 3.89E-05 | 0.000204802 |
| LINC01532 | 3.076446489 | 1.706537556 | -0.850192734 | 4.46E-09 | 8.37E-08 |
| LINC02298 | 0.572054968 | 0.346126792 | -0.724853163 | 2.62E-06 | 2.04E-05 |
| AC061961.1 | 1.246362098 | 0.58149971 | -1.099872889 | 5.22E-07 | 5.29E-06 |
| AC110015.1 | 0.268027522 | 0.544659673 | 1.022973902 | 1.12E-06 | 9.92E-06 |
| AC091932.1 | 0.285138773 | 0.740862612 | 1.377541799 | 2.42E-06 | 1.93E-05 |
| AC135782.1 | 0.683360615 | 0.413660604 | -0.724199534 | 1.09E-06 | 9.80E-06 |
| AC244517.6 | 0.310359636 | 0.502140999 | 0.694151581 | 0.000181485 | 0.000781719 |
| MIR4435-2HG | 0.516668793 | 1.081763027 | 1.066072842 | 8.51E-11 | 2.84E-09 |
| FIRRE | 0.422532533 | 0.767696155 | 0.861472997 | 2.97E-08 | 4.34E-07 |
| AC148477.1 | 1.02854946 | 0.644001424 | -0.675475388 | 0.000144786 | 0.000642237 |
| AC010201.1 | 0.433686336 | 0.672884151 | 0.633706151 | 0.00014655 | 0.000648575 |
| AL365259.1 | 1.214213589 | 0.713322159 | -0.767396529 | 1.98E-11 | 8.53E-10 |
| SOCS2-AS1 | 0.564322691 | 1.091939908 | 0.952301199 | 0.008502563 | 0.019620951 |
| AC013553.4 | 0.35584572 | 0.53973785 | 0.601006976 | 0.004238855 | 0.011048443 |
| AC064875.1 | 0.269906847 | 1.12758335 | 2.062700599 | 9.58E-08 | 1.19E-06 |
| AJ011932.1 | 0.332752508 | 0.872695717 | 1.391029174 | 5.75E-10 | 1.50E-08 |
| AC093673.1 | 4.799155519 | 8.036316323 | 0.743753787 | 3.80E-17 | 1.84E-14 |
| AC010173.1 | 0.380060837 | 0.598720422 | 0.65565211 | 1.31E-12 | 9.17E-11 |
| AL133415.1 | 0.281415843 | 0.560431737 | 0.993835108 | 2.27E-06 | 1.83E-05 |
| DCTN1-AS1 | 0.535975864 | 0.319810836 | -0.74494921 | 0.024086889 | 0.047054589 |
| ANKRD62P1-PARP4P3 | 0.776817641 | 0.330066655 | -1.234818565 | 2.04E-09 | 4.24E-08 |
| AP000553.2 | 0.525392393 | 0.86458655 | 0.718615081 | 5.80E-07 | 5.69E-06 |
| AL138767.3 | 0.814245347 | 0.340586276 | -1.257443267 | 3.59E-14 | 4.62E-12 |
| MRVI1-AS1 | 0.502909646 | 0.333866156 | -0.59102937 | 1.03E-09 | 2.54E-08 |
| WARS2-IT1 | 0.593907407 | 0.917167328 | 0.626946939 | 2.19E-06 | 1.79E-05 |
| LNCTAM34A | 0.412826222 | 0.660759354 | 0.678590331 | 2.55E-06 | 2.01E-05 |
| CRNDE | 0.858686285 | 2.468971771 | 1.523707288 | 6.96E-15 | 1.12E-12 |
| AC010273.2 | 0.468871732 | 0.959780903 | 1.033511805 | 3.97E-10 | 1.11E-08 |
| AC018410.1 | 0.717255389 | 0.337371174 | -1.088150191 | 2.08E-08 | 3.21E-07 |
| LINC02427 | 1.029553097 | 0.665760862 | -0.628942269 | 1.23E-11 | 5.80E-10 |
| AC124303.1 | 0.672986665 | 0.446415361 | -0.592191248 | 0.000192886 | 0.000825314 |
| AC008759.2 | 0.758205745 | 0.451981407 | -0.746325961 | 5.47E-13 | 4.60E-11 |
| AC104088.1 | 0.564923834 | 0.292407271 | -0.950077181 | 6.36E-11 | 2.20E-09 |
| LINC01831 | 0.154674657 | 0.734951028 | 2.248411286 | 2.08E-13 | 2.01E-11 |
| AL355974.3 | 1.528651129 | 3.485717079 | 1.189196287 | 2.90E-08 | 4.28E-07 |
| AC124854.1 | 1.705458071 | 0.820496883 | -1.055589528 | 6.39E-18 | 4.12E-15 |
| LINC01956 | 0.164126222 | 0.827915691 | 2.33467811 | 2.35E-21 | 4.55E-18 |
| AC026790.1 | 0.813234407 | 0.458545569 | -0.826606144 | 1.34E-05 | 8.23E-05 |
| AL158847.1 | 0.920479016 | 0.558609901 | -0.720543688 | 5.11E-09 | 9.50E-08 |
| AC008760.2 | 0.566407971 | 0.88993271 | 0.651854687 | 0.000868994 | 0.00294848 |
| AC092718.4 | 2.145993574 | 3.413262957 | 0.669505808 | 2.32E-11 | 9.74E-10 |
| LINC01785 | 0.488440669 | 0.317294391 | -0.622361311 | 0.017957891 | 0.036674299 |
| AL121821.1 | 0.985791012 | 0.48790759 | -1.014673901 | 1.29E-10 | 4.03E-09 |
| BASP1-AS1 | 0.701213221 | 0.351060809 | -0.998132247 | 1.74E-09 | 3.91E-08 |
| AC136475.2 | 5.172671759 | 3.274596375 | -0.659592554 | 1.90E-09 | 4.11E-08 |
| AF131216.3 | 3.444527712 | 2.27858198 | -0.596169907 | 1.79E-09 | 3.93E-08 |
| AC110491.1 | 0.994653514 | 0.643616852 | -0.627991951 | 0.003072236 | 0.008416013 |
| AL357992.1 | 0.573585378 | 0.974559981 | 0.764742736 | 1.35E-05 | 8.29E-05 |
| AC005220.1 | 0.750061602 | 0.498871394 | -0.588341143 | 9.34E-06 | 5.98E-05 |
| AC131097.4 | 0.223188939 | 0.693529505 | 1.63569173 | 0.001095698 | 0.003525925 |
| AC103724.4 | 0.333562132 | 0.514337712 | 0.624760423 | 0.009644859 | 0.021816559 |
| AC021739.3 | 2.292331789 | 1.376148845 | -0.736179352 | 9.33E-08 | 1.17E-06 |
| AL137025.1 | 1.146822358 | 0.634119343 | -0.854815646 | 7.23E-12 | 3.59E-10 |
| HOXD-AS2 | 0.298261436 | 0.954925811 | 1.678811197 | 7.01E-17 | 2.71E-14 |
| AP001972.1 | 0.52688892 | 0.319105533 | -0.723465217 | 6.94E-07 | 6.64E-06 |
| AC090692.1 | 1.248997406 | 2.308332325 | 0.88608046 | 3.24E-10 | 9.25E-09 |
| AC016168.2 | 0.26112513 | 0.558021129 | 1.095578445 | 2.93E-07 | 3.16E-06 |
| AC138649.1 | 1.549780347 | 1.017502975 | -0.607030742 | 0.00121095 | 0.003839305 |
| MPPED2-AS1 | 0.401218724 | 0.628475488 | 0.647467541 | 0.000966126 | 0.003194037 |
| RNF219-AS1 | 1.830454828 | 0.897092542 | -1.028873449 | 1.01E-15 | 1.96E-13 |
| AL049871.1 | 0.167635679 | 0.676831995 | 2.013468532 | 5.88E-09 | 1.06E-07 |
| AC015712.2 | 0.570361395 | 0.313488263 | -0.863464908 | 0.000197129 | 0.00083975 |
| AC097641.1 | 0.577981178 | 0.24999439 | -1.20912679 | 4.45E-12 | 2.48E-10 |
| AP002840.2 | 0.35566619 | 0.599532117 | 0.753313205 | 0.000627352 | 0.002214051 |
| AC002451.1 | 0.921070674 | 0.521418764 | -0.820869358 | 8.78E-11 | 2.86E-09 |
| AL390786.1 | 0.848419028 | 0.409248847 | -1.051798625 | 3.35E-12 | 2.03E-10 |
| AL356056.2 | 1.250541076 | 0.776165344 | -0.688116524 | 8.63E-13 | 6.42E-11 |
| INSYN1-AS1 | 1.453472284 | 0.931902299 | -0.641252945 | 2.04E-07 | 2.29E-06 |
| AC026401.3 | 2.102195216 | 3.947184126 | 0.90892717 | 1.58E-14 | 2.35E-12 |
| AP005432.2 | 0.282242385 | 0.563231932 | 0.996794475 | 1.38E-07 | 1.65E-06 |
| LINC01018 | 0.689758742 | 0.401244808 | -0.781609114 | 0.0033307 | 0.008971551 |
| LINC02058 | 1.587439987 | 0.6171268 | -1.3630632 | 3.27E-16 | 9.04E-14 |
| AP001025.1 | 0.395669473 | 0.66610002 | 0.751443063 | 0.000292732 | 0.001179468 |
| LDLRAD4-AS1 | 0.641490127 | 0.367912319 | -0.802065078 | 6.23E-06 | 4.23E-05 |
| AC087623.1 | 0.500527927 | 0.789679091 | 0.657815924 | 0.017181515 | 0.035577142 |
| AC104024.2 | 0.780439235 | 0.338926538 | -1.203313701 | 0.006979962 | 0.016624687 |
| AC131571.1 | 0.679566316 | 0.387648844 | -0.809863981 | 4.75E-10 | 1.31E-08 |
| AC007098.1 | 0.806994237 | 1.230818493 | 0.608987749 | 2.04E-09 | 4.24E-08 |
| NEBL-AS1 | 1.312306253 | 0.856288582 | -0.615935448 | 4.82E-10 | 1.31E-08 |
| LINC00327 | 0.339102538 | 0.611498445 | 0.85062725 | 5.22E-10 | 1.40E-08 |
| TGFB2-AS1 | 1.65577332 | 3.214408858 | 0.957048268 | 6.73E-09 | 1.18E-07 |
| AC092849.1 | 0.333275512 | 0.513610535 | 0.623959477 | 1.03E-05 | 6.52E-05 |
| LINC01007 | 0.740307864 | 0.168017534 | -2.139513559 | 0.00094112 | 0.003131162 |
| AC002454.1 | 0.160563184 | 0.654201655 | 2.026594281 | 0.001420204 | 0.004352893 |
| NBAT1 | 0.565548484 | 0.872332943 | 0.625228163 | 0.000231996 | 0.00095872 |
| LINC01579 | 0.401522107 | 1.311421536 | 1.707580164 | 1.01E-05 | 6.41E-05 |
| AL121956.4 | 0.937556353 | 0.463794518 | -1.015419641 | 8.14E-11 | 2.76E-09 |
| AC138474.1 | 0.527075418 | 0.831774888 | 0.65818372 | 1.59E-07 | 1.85E-06 |
| AC092675.1 | 0.765268356 | 1.183857158 | 0.629457369 | 1.09E-05 | 6.78E-05 |
| SNHG18 | 0.884871879 | 1.522062225 | 0.782486854 | 0.000131721 | 0.000593821 |
| AL021807.1 | 0.42102961 | 0.767601163 | 0.866435201 | 2.49E-06 | 1.98E-05 |
| AC012073.1 | 0.805142797 | 1.252105852 | 0.637039949 | 2.55E-11 | 1.05E-09 |
| AL031056.1 | 4.06673219 | 2.434189889 | -0.740428271 | 6.89E-05 | 0.000335879 |
| LINC01338 | 4.391967953 | 2.483624816 | -0.822420275 | 1.46E-08 | 2.35E-07 |
| AC062021.1 | 4.985896895 | 3.250846212 | -0.617037741 | 1.50E-07 | 1.78E-06 |
| LINC01088 | 6.994522548 | 4.185664458 | -0.740768918 | 3.78E-07 | 4.00E-06 |
| LBX2-AS1 | 0.618257816 | 0.942622671 | 0.608471806 | 1.40E-08 | 2.30E-07 |
| AC007666.1 | 1.267122451 | 0.840007587 | -0.593081685 | 1.43E-08 | 2.32E-07 |
| AL512785.1 | 0.339901773 | 0.930142715 | 1.452334202 | 3.45E-05 | 0.000185065 |
| AC103563.7 | 0.814467515 | 0.516514467 | -0.6570484 | 0.000110025 | 0.000509065 |
| AC044839.1 | 2.468899352 | 1.588875 | -0.635862395 | 2.77E-11 | 1.12E-09 |
| SLCO4A1-AS1 | 0.318637509 | 0.718064334 | 1.172196999 | 0.013216362 | 0.028591101 |
| AC015540.1 | 2.857060133 | 1.833870707 | -0.639639473 | 1.99E-10 | 6.12E-09 |
| AC097381.3 | 0.805019452 | 0.533306104 | -0.594059804 | 0.001309469 | 0.004065028 |
| AC108156.1 | 0.852130107 | 0.551467155 | -0.627798763 | 6.15E-07 | 5.95E-06 |
| RFPL1S | 2.503904934 | 1.376760013 | -0.862902688 | 1.44E-06 | 1.23E-05 |
| AC136475.9 | 1.054062979 | 0.634679916 | -0.731859973 | 1.56E-07 | 1.84E-06 |
| FOXD3-AS1 | 0.690153283 | 2.027998208 | 1.555067652 | 5.27E-11 | 1.92E-09 |
| EMX2OS | 2.968554797 | 1.916416007 | -0.631349976 | 5.27E-06 | 3.74E-05 |
| AC009227.1 | 1.185926695 | 0.769701017 | -0.623644778 | 6.11E-08 | 8.20E-07 |
| AF106564.1 | 1.348990861 | 0.805269601 | -0.744336796 | 4.46E-08 | 6.34E-07 |
| HAR1A | 1.619127665 | 1.075502155 | -0.590206329 | 5.98E-06 | 4.10E-05 |
| ISX-AS1 | 1.02147721 | 0.414218618 | -1.302192711 | 1.43E-09 | 3.44E-08 |
| AC021242.3 | 0.890474415 | 1.361509709 | 0.612561205 | 0.007130839 | 0.016880101 |
| AC091057.1 | 0.291361296 | 0.59339159 | 1.026175235 | 2.57E-14 | 3.55E-12 |
| PANTR1 | 11.26310036 | 18.07596206 | 0.682468425 | 2.09E-16 | 6.73E-14 |
| AC211476.2 | 0.317010776 | 0.488209911 | 0.622969703 | 1.80E-07 | 2.07E-06 |
| AP000696.1 | 0.338403988 | 0.750695443 | 1.149481154 | 5.88E-08 | 8.01E-07 |
| LINC02593 | 2.252720244 | 1.448749586 | -0.636859914 | 9.01E-07 | 8.30E-06 |
| AC009318.1 | 0.520865776 | 0.783789687 | 0.589554943 | 1.62E-07 | 1.87E-06 |
| LINC00836 | 3.672622496 | 1.7975714 | -1.030761535 | 1.68E-12 | 1.12E-10 |
| AL391834.1 | 3.416126419 | 2.12066271 | -0.687846186 | 9.44E-14 | 1.07E-11 |
| AC109439.2 | 2.395596544 | 0.644302714 | -1.89457438 | 7.50E-20 | 7.25E-17 |
| CYTOR | 0.588186334 | 1.17573678 | 0.999219941 | 1.19E-08 | 1.97E-07 |
| DDX11-AS1 | 0.332150486 | 0.534565174 | 0.686528827 | 1.66E-08 | 2.66E-07 |
| AC002456.1 | 1.341528782 | 2.276748117 | 0.763096683 | 1.09E-13 | 1.17E-11 |
| UFL1-AS1 | 0.562996759 | 0.360254534 | -0.64411003 | 1.03E-07 | 1.28E-06 |
| LINC02217 | 0.667878526 | 0.240895075 | -1.471180832 | 0.000569736 | 0.002040499 |
| AC009118.1 | 0.890066786 | 0.424449836 | -1.068319535 | 2.72E-09 | 5.48E-08 |
| AL354919.2 | 0.644095316 | 1.276327964 | 0.986652985 | 3.29E-09 | 6.54E-08 |
| LINC01998 | 0.643011671 | 0.373845823 | -0.782401508 | 2.17E-08 | 3.30E-07 |
| AL512329.2 | 0.912191262 | 1.497010191 | 0.714675786 | 0.025849635 | 0.049893407 |
| PVT1 | 0.409029212 | 0.701324214 | 0.777877659 | 5.96E-06 | 4.10E-05 |
| LINC02587 | 0.315083273 | 2.130637786 | 2.75748028 | 0.005777141 | 0.014178921 |
| AC026333.4 | 0.330582639 | 0.583640629 | 0.820069354 | 1.99E-06 | 1.63E-05 |
| AC021683.1 | 0.863427521 | 0.545499867 | -0.662496234 | 0.017328128 | 0.035727718 |
| VSTM2A-OT1 | 0.654750504 | 0.431423122 | -0.601841766 | 3.16E-06 | 2.45E-05 |
| AL139412.1 | 0.38916714 | 0.602908882 | 0.631550083 | 8.79E-08 | 1.12E-06 |
| AC025171.5 | 0.269306354 | 0.850236503 | 1.658615928 | 5.16E-09 | 9.50E-08 |
| DNMBP-AS1 | 1.066865422 | 0.40163067 | -1.409436853 | 8.01E-16 | 1.94E-13 |
| AL450311.1 | 1.950384069 | 1.253647238 | -0.6376268 | 2.61E-07 | 2.86E-06 |
| AC017104.1 | 0.573136762 | 1.111617897 | 0.955709626 | 1.75E-15 | 3.07E-13 |
| AC004067.1 | 0.69635291 | 1.052450876 | 0.595862345 | 0.002441384 | 0.006923221 |
